# Targeting FDFT1 Reduces Cholesterol and Bile Acid Production and Delays Hepatocellular Carcinoma Progression Through the HNF4A/ALDOB/AKT1 Axis

**DOI:** 10.1002/advs.202411719

**Published:** 2025-02-03

**Authors:** Dong Cai, Guo‐Chao Zhong, Xin Dai, Zhibo Zhao, Menglin Chen, Jiejun Hu, Zhenru Wu, Lve Cheng, Shengwei Li, Jianping Gong

**Affiliations:** ^1^ Department of Hepatobiliary Surgery The Second Affiliated Hospital of Chongqing Medical University Chongqing 400010 China; ^2^ Institute of Clinical Pathology Key Laboratory of Transplant Engineering and Immunology NHC West China Hospital Sichuan University Chengdu Sichuan 610041 China

**Keywords:** bile acid, cholesterol, FDFT1, hepatocellular carcinoma, proliferation

## Abstract

Targeting cholesterol metabolism is a novel direction for tumor therapy. Unfortunately, the current use of statins for hepatocellular carcinoma (HCC) is controversial. Herein, farnesyl‐diphosphate farnesyltransferase 1 (FDFT1) is identified as a novel target for treating HCC and a potential alternative to statins. Twenty‐three key genes in cholesterol biosynthesis are screened, and FDFT1 is identified via public databases (The Cancer Genome Atlas, International Cancer Genome Consortium and Gene Expression Omnibus). Clinical samples reveal that FDFT1 is highly expressed in HCC tissues, and this phenotype is strongly associated with a poor prognosis. Functionally, FDFT1 knockdown inhibits the proliferation and metastasis of HCC cells and suppresses hepatocarcinogenesis in vitro and in vivo, whereas FDFT1 overexpression promotes HCC cell proliferation and metastasis. Mechanistically, FDFT1 downregulation decreases cholesterol and bile acid levels and then increases hepatocyte nuclear factor 4 alpha (HNF4A) transcriptional activity. Experiments indicate that HNF4A combines with the promoter of aldolase B (*ALDOB)* and promotes the ALDOB transcription and that ALDOB combines with AKT serine/threonine kinase 1 (AKT1) and inhibits AKT1 phosphorylation. Moreover, FDFT1 knockdown combined with AKT inhibitor (AZD5363) treatment shows remarkable therapeutic potential. FDFT1 inhibition reduces cholesterol and bile acid levels to delay HCC progression through the HNF4A/ALDOB/AKT1 axis. Thus, targeting FDFT1 may be a novel potential strategy for treating HCC.

## Graphical abstract

Farnesyl‐diphosphate farnesyltransferase 1 (FDFT1) synthesizes squalene and participates in cholesterol biosynthesis. This study demonstrates the essential role of FDFT1 in cholesterol metabolism and HCC development. In vivo and in vitro, FDFT1 knockdown downregulates cholesterol and total bile acid levels and inhibits HCC proliferation and metastasis. Moreover, the regulation of FDFT1 on HCC depends on the HNF4A/ALDOB/AKT1 axis.

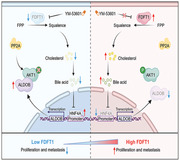

## 1 Introduction

Liver cancer is the third most common cause of cancer‐related death and ranks sixth among cancers in terms of incidence worldwide.^[^ ^1^ ^]^ Hepatocellular carcinoma (HCC) is the most common type of liver cancer, accounting for ≈90% of cases.^[^ ^2^ ^]^ Recently, glucose and lipid metabolism disorders have rapidly become acknowledged as common etiological factors of HCC.^[^ ^2^ ^]^ Notably, the incidence of complications in patients with metabolic disorders‐related HCC receiving resection is more than 30%, similar to that observed in patients with cirrhosis.^[^ ^3^ ^]^ Despite recent advances in targeted therapy for unresectable HCC, the median overall survival (OS) is still <2 years.^[^ ^4^ ^]^ Thus, the development of more targeted therapeutic strategies for HCC, especially for unresectable HCC, is urgently needed.

The liver is the core organ regulating metabolism, and hepatocytes are the main site of cholesterol biosynthesis.^[^ ^5^ ^]^ Many solid tumors undergo metabolic reprogramming, an important hallmark of cancer.^[^ ^6^ ^]^ Cholesterol metabolism is activated in HCC and has been shown to facilitate HCC progression.^[^ ^7^ ^]^ Importantly, preclinical studies have shown that inhibiting cholesterol metabolism impairs HCC development,^[^ ^8^ ^]^ supporting the theoretical feasibility of targeting cholesterol metabolism for HCC treatment. Currently, statins are the main drugs used to lower cholesterol in clinical practice.^[^ ^9^ ^]^ However, the use of statins for HCC treatment is still controversial. On the one hand, several clinical studies have shown that statin treatment fails to improve the prognosis of HCC.^[^ ^10^ ^]^ On the other hand, some HCC patients have several adverse reactions (such as muscle symptoms and impaired liver function).^[^ ^11^ ^]^ Thus, more studies are needed to identify novel cholesterol metabolism targets for HCC treatment. Farnesyl‐diphosphate farnesyltransferase 1 (FDFT1) synthesizes squalene and participates in cholesterol biosynthesis. Interestingly, FDFT1 inhibits tumor growth in colorectal and gastric cancers but promotes tumor growth in squamous cell carcinoma of the tongue and pancreatic cancer.^[^ ^12^ ^]^ However, the roles of FDFT1 in HCC are still unclear.

Here, we found that FDFT1 expression is upregulated and strongly associated with a poor prognosis in HCC patients. Functionally, FDFT1 knockdown inhibited tumor growth and metastasis, whereas FDFT1 overexpression promoted tumor growth and metastasis in vivo and in vitro. Mechanistically, FDFT1 knockdown decreases cholesterol and bile acid levels in HCC cells and promotes hepatocyte nuclear factor 4 alpha (HNF4A) transcriptional activity. HNF4A subsequently combines with the promoter of aldolase B (*ALDOB)* and promotes ALDOB transcription. Furthermore, ALDOB combines with AKT serine/threonine kinase 1 (AKT1) and inhibits AKT1 phosphorylation. Additionally, FDFT1 inhibition combined with AKT inhibitor (AZD5363) treatment showed promising therapeutic efficacy in treating HCC.

## 2 Results

### 2.1 FDFT1 is Highly Expressed in HCC and Indicates a Poor Prognosis

**Figure 1:**
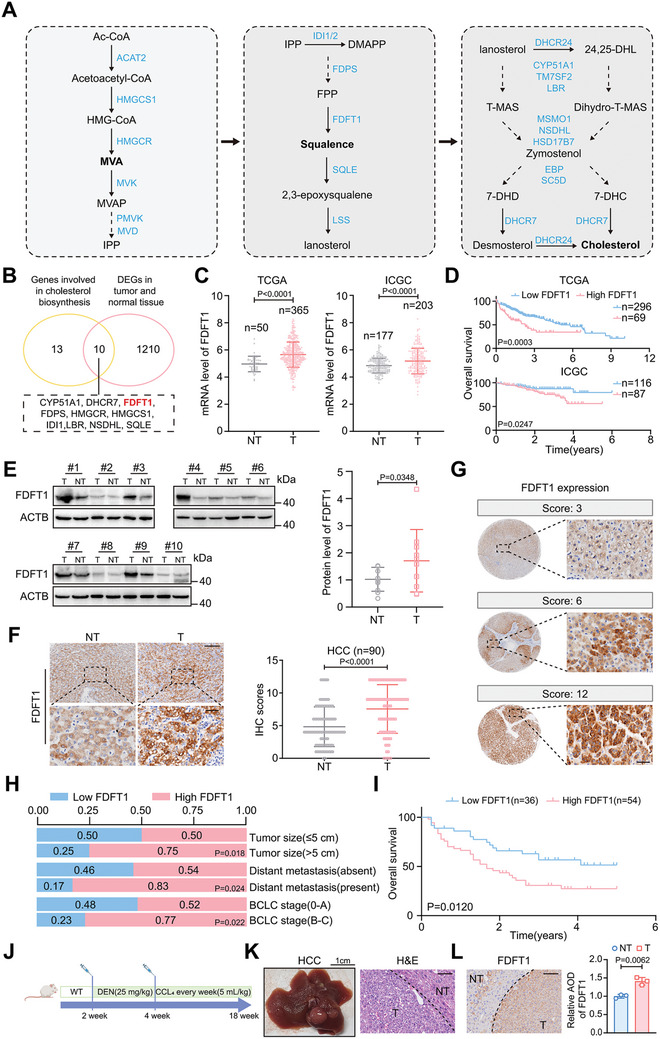
FDFT1 is highly expressed in HCC and tightly associated with tumor prognosis. A) Cholesterol de novo synthesis pathway in cells. B) Venn plot of cholesterol biosynthesis gene sets and DEGs between HCC and normal tissues from the integrated GEO dataset. C) FDFT1 mRNA levels in T and NT from TCGA and ICGC datasets. D) Kaplan‐Meier survival curves of FDFT1 high and low expression grouping in TCGA and ICGC cohorts. The cutoff values were determined by R. E) Western blot from 10 pairs of fresh‐frozen HCC tumors and paired adjacent non‐tumor tissues. *n* = 10. F) Representative images (left panel) and statistical analysis (right panel) of FDFT1 IHC from 90 HCC patients. Scale bars, 200 (upper panel) and 40 (lower panel) µm. G) Representative images of different FDFT1 IHC scores. Scale bars, 40 µm. H) Comparison of clinical features between high and low FDFT1 groups. I) Kaplan‐Meier survival curves of 90 HCC patients based on IHC scores. J) Schematic diagram of the construction of DEN/CCL4 induced HCC mouse models. K) Images showing the liver morphology of HCC mice. Scale bars, 1 cm. L) Representative images of FDFT1 staining in HCC mice. Images were quantified by the image J. *n* = 3. Scale bars, 100 µm. Data are presented as mean ± SD (C, E‐right panel, F‐right panel, L‐right panel). Data were analyzed by paired t‐test (E‐right panel, L‐right panel), Mann‐Whitney U test (C, F‐right panel), and Chi‐square test (H). DEGs, differentially expressed genes; T, tumor tissue; NT, non‐tumor tissue; AOD, average optical density. Image (J) created with BioRender.com. with permission.

Cholesterol biosynthesis metabolism has been found to be upregulated in HCC and to facilitate tumor progression.^[^ ^7^ ^]^ Consistent with the above studies, gene set enrichment analysis (GSEA) based on RNA sequencing (RNA‐seq) datasets confirmed that the cholesterol biosynthesis pathway was enriched in HCC (Figure S1A,B, Supporting Information). Although the mechanisms regulating cholesterol biosynthesis are complex, only 23 key genes participate in de novo cholesterol synthesis (**Figure** **1**A). To identify the key molecules in these genes, we first identified 1220 differentially expressed genes (DEGs) between tumor and nontumor tissues based on an integrated dataset of 733 HCC patients from the Gene Expression Omnibus (GEO) database (Figure S1C,D, Supporting Information) and then intersected the cholesterol biosynthesis‐related gene set with the abovementioned DEG dataset, yielding a gene dataset consisting of 10 genes (Figure 1B). Next, univariate Cox analysis was used to reduce the number of candidate genes (Figure S1E–G, Supporting Information). The genes were ranked according to the expression levels in HCC, and we found that only FDFT1 ranked in the top two in both datasets (Figure S1H,I, Supporting Information). Thus, we chose FDFT1 as our research focus.

We found that FDFT1 was highly expressed in HCC at the RNA level and that this phenotype indicated a poor prognosis (Figure 1C,D). Then, we assessed FDFT1 protein level in HCC tissues and paired adjacent nontumor tissues (*n* = 10 per group). Consistently, the FDFT1 protein level in HCC tissues was also higher than that in nontumor tissues (Figure 1E). To further assess FDFT1 expression in HCC, we compared the immunohistochemistry (IHC) scores for FDFT1 expression in 90 HCC tissues and paired adjacent nontumor tissues and obtained consistent results (Figure 1F). Interestingly, we found that FDFT1 expression was heterogeneous in tumor tissues, although it was generally expressed at higher levels in tumors than in nontumor tissues (Figure 1G). Moreover, we found that FDFT1 expression was positively correlated with tumor size, distant metastasis, and Barcelona Clinic Liver Cancer (BCLC) (Figure 1H) and negatively correlated with overall survival (Figure 1I). In addition, we constructed a DEN/CCL_4_‐ induced HCC mouse model and found that FDFT1 expression was obviously higher in tumor tissues than in nontumor tissues (Figure 1J–L). These observations indicate that FDFT1 may play an important role in HCC and serve as an unfavorable prognostic biomarker for HCC.

### 2.2 FDFT1 Promotes HCC Proliferation and Metastasis In Vitro and Vivo

To further explore the effect of FDFT1 on HCC, we first measured the effect of FDFT1 on the proliferation and metastasis of HCC cells. Considering the heterogeneity of FDFT1 expression in HCC cells, we first analyzed the *FDFT1* mRNA and protein levels in various HCC cells (Figure S2A,B, Supporting Information). According to the FDFT1 expression in various HCC cells, we knocked down FDFT1 in Huh7 and HCCLM3 cells and overexpressed FDFT1 in Hep3B cells (Figure S2C,D, Supporting Information). CCK8, colony formation, and 5‐ethynyl‐2′‐deoxyuridine (EdU) labeling assays suggested that FDFT1 knockdown suppressed the proliferation of HCC cells, whereas FDFT1 overexpression promoted their proliferation (Figure S2E–K, Supporting Information). Moreover, migration and invasion assays indicated that FDFT1 downregulation impaired metastasis of HCC cells, whereas FDFT1 upregulation had the opposite effect (Figure S3A–C, Supporting Information). Since epithelial‐mesenchymal transition (EMT) is an important mechanism responsible for tumor cell metastasis,^[^ ^13^ ^]^ we detected the protein levels of EMT‐related markers in HCC cells. As expected, E‐cadherin was upregulated and vimentin was downregulated after FDFT1 knockdown, while FDFT1 overexpression induced the opposite effect (Figure S3D–G, Supporting Information).

**Figure 2:**
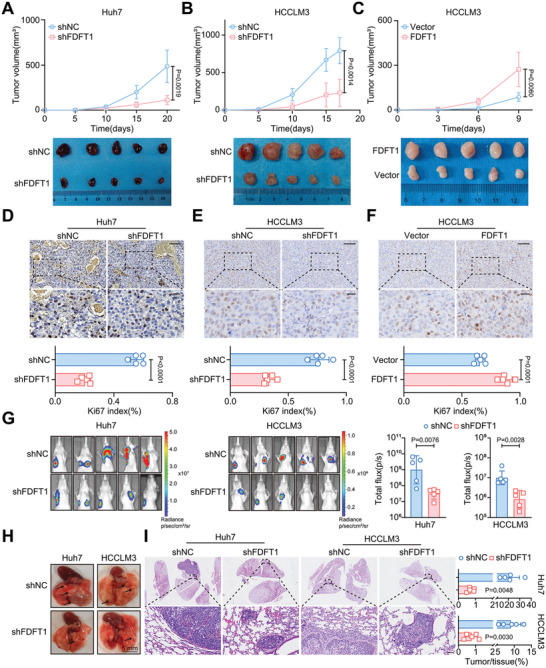
FDFT1 knockdown inhibits proliferation and metastasis of HCC in vivo. A–C) Tumor volume (upper panel) and images of tumors harvested at the endpoint (lower panel) of FDFT1 knockdown (A‐B) or overexpression (C) in subcutaneous xenograft models. *n* = 5. D‐F) Representative images of Ki67 IHC (upper panel) and statistical analysis (lower panel) of FDFT1 knockdown (D‐E) or overexpression (F) in subcutaneous xenograft models. Ki67 index (%) was completed by image J. Scale bars, 100 (upper panel) and 25 (lower panel) µm. *n* = 5. G) Bioluminescence images of lung metastasis (left panel) and statistical analysis (right panel) of FDFT1 knockdown in lung metastasis models. *n* = 5. H‐I) Representative images of lung morphology (H), H&E staining (I‐left panel), and statistical analysis (I‐right panel) of FDFT1 knockdown in lung metastasis models. Scale bars, 5 mm (H) and 50 µm (I). *n* = 5. All Data are presented as mean ± SD. Data were analyzed by two‐way ANOVA with Bonferroni multiple‐comparison correction (A‐C), unpaired t‐test (D‐G), and unpaired t‐test with Welch correction (I). H&E, hematoxylin‐eosin.

**Figure 3:**
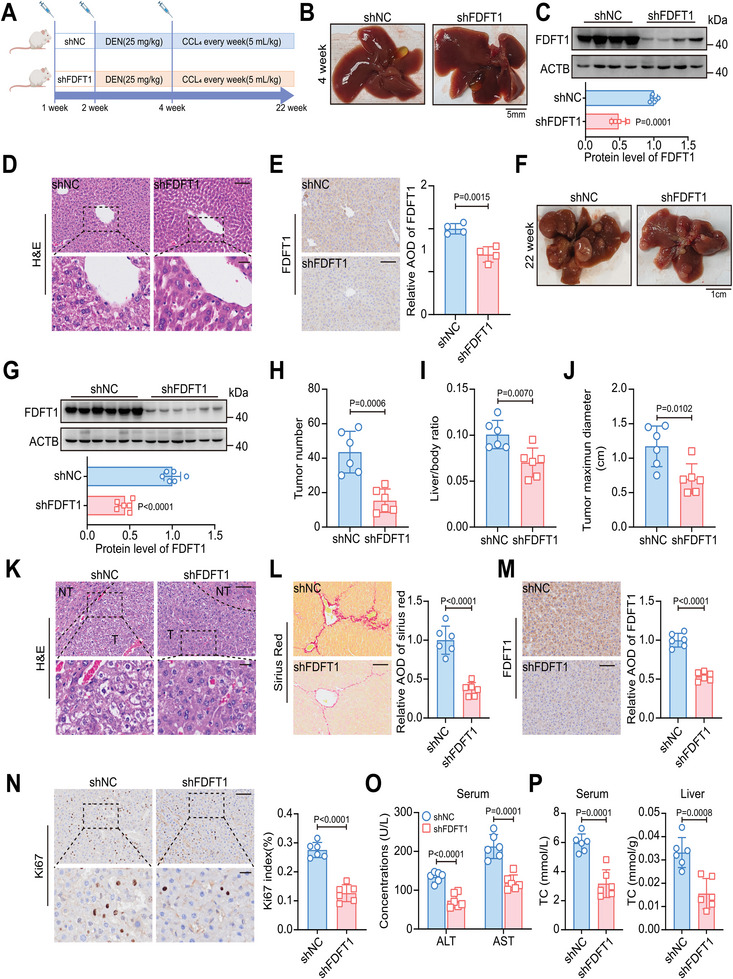
Liver‐specific FDFT1 knockdown delays HCC progression. A) Schematic diagram of the construction of DEN/CCL4 induced HCC mouse models combined with or without AAV8‐mediated FDFT1 knockdown. B) Images showing the liver morphology of 4‐week‐old mice in shNC and shFDFT1 groups. Scale bars, 5 mm. C) FDFT1 protein levels in liver tissues of 4‐week‐old mice from shNC and shFDFT1 groups. *n* = 4. D‐E) Representative images of H&E and FDFT1 staining in liver tissues of 4‐week‐old mice from shNC and shFDFT1 groups. Scale bars, 100 (D‐upper panel, E) and 25 (D‐lower panel) µm. *n* = 4. F) Images showing the liver morphology of mice in shNC and shFDFT1 groups. Scale bars, 1 cm. G) FDFT1 protein levels in liver tissues of 22‐week‐old mice from shNC and shFDFT1 groups. *n* = 6. H‐J) Comparison of tumor number (H), liver/body ratio (I), and tumor maximum diameter (J) between shNC and shFDFT1 groups. *n* = 6. K‐M) Representative images of H&E, Sirius, and FDFT1 staining in liver tumor tissues of 22‐week‐old mice from shNC and shFDFT1 groups. Scale bars, 100 (K‐upper panel, L‐M) and 25 (K‐lower panel) µm. *n* = 6. N) Representative images of Ki67 IHC (left panel) of samples for liver cancer models and statistical analysis (right panel). The Ki67 index (%) was completed by image J. Scale bars, 100 (upper panel) and 25 (lower panel) µm. *n* = 6. O‐P) Detection of ALT and AST levels in the serum (O), TC in serum, and liver (P) from HCC mice. *n* = 6. Data are presented as mean ± SD (C, E, G‐J, L‐M, N‐right panel, and O‐P). Images were quantified by image J (C, E, G, and L–N). Data were analyzed by unpaired t‐tests (C, E, G, H‐J, L‐N‐right panel, and O‐P). H&E, hematoxylin‐eosin; ALT, alanine aminotransferase; AST, aspartate aminotransferase; TC, total cholesterol; HCC, hepatocellular carcinoma; AOD, average optical density. Image (A) created with BioRender.com. with permission.

Furthermore, in tumor xenograft models, FDFT1 inhibition significantly impaired tumor formation and reduced tumor weight (**Figure** **2**A,B; Figure S4A, Supporting Information), whereas FDFT1 overexpression increased tumor volume and weight (Figure 2C; Figure S4B, Supporting Information). Consistently, Ki67 staining confirmed that FDFT1 enhanced HCC proliferation in vivo (Figure 2D–F; Figure S4C–D, Supporting Information). Additionally, FDFT1 downregulation decreased the fluorescence intensity and number of lung lesions in vivo (Figure 2G–I). Then, we constructed liver‐ specific FDFT1‐knockdown HCC mice via AAV8 with the TBG promoter (**Figure** **3**A). The results of immunohistochemical (IHC) and western blotting demonstrated that tail vein injection of AAV8‐shFDFT1 successfully knocked down FDFT1 expression in the liver (Figure 3B–E). Notably, FDFT1 knockdown was still effective at 22 weeks and significantly relieved the tumor burden, as evidenced by the decreases in tumor number, liver/body ratio, and tumor maximum diameter in the FDFT1‐knockdown group (Figure 3F–K). Sirius Red and Ki67 staining also revealed that FDFT1 knockdown suppressed the proliferation of tumor cells (Figure 3L–N). Moreover, FDFT1 knockdown improved liver function by reducing alanine aminotransferase (ALT) and aspartate aminotransferase (AST) levels in the serum (Figure 3O). Importantly, FDFT1 knockdown in vivo decreased cholesterol levels in the liver and serum (Figure 3P). Overall, these results indicate that FDFT1 plays a key role in HCC development and cholesterol metabolism.

### 2.3 FDFT1 Inhibition Upregulates ALDOB to Weaken AKT1 Phosphorylation in HCC

**Figure 4:**
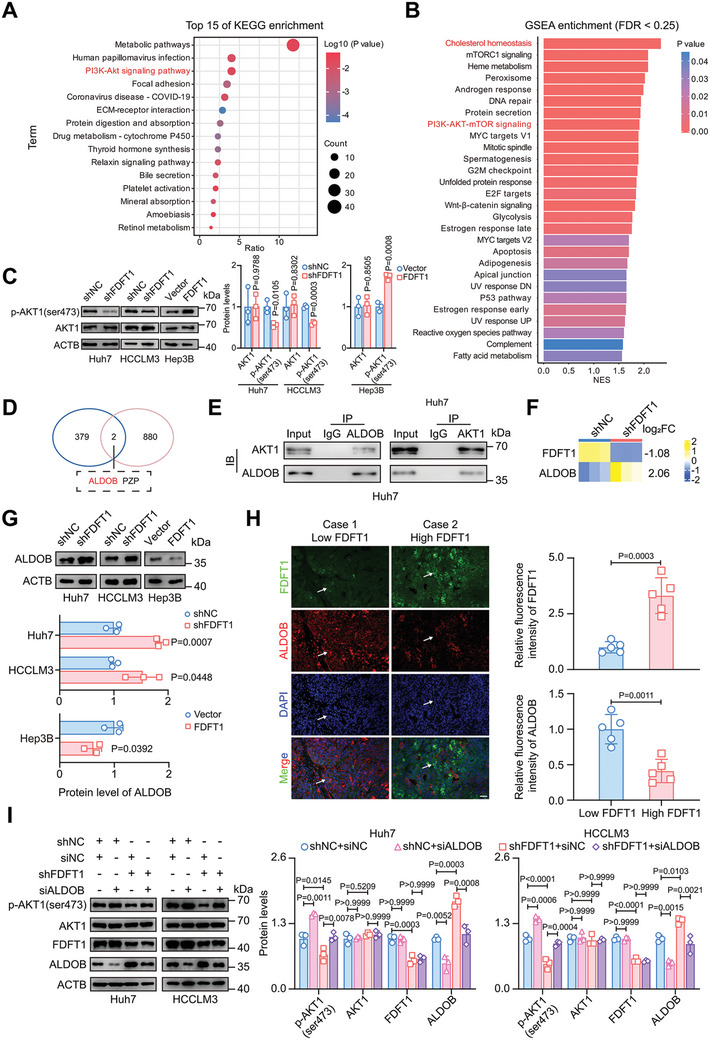
FDFT1 knockdown impairs AKT1 phosphorylation via ALDOB to inhibit HCC. A) Bubble diagram of top 15 KEGG results from DEGs between shFDFT1 and shNC groups in Huh7 cells through the DAVID website. B) GSEA results with FDR < 0.25 of high and low FDFT1 expression groups in the TCGA cohort. C) Western blot of total AKT1 and phosphorylated AKT1 (ser473) in FDFT1 knockdown or overexpression HCC cells. *n* = 3. D) Venn plot of DEGs between shFDFT1 and shNC groups in Huh7 cells and captured proteins interacted with AKT1 through mass spectrometry. E) Co‐IP results show that ALDOB interacts with AKT1 in Huh7 cells. F) Fold change of ALDOB after FDFT1 knockdown in Huh7 cells. *n* = 3. G) Western blot of ALODB in FDFT1 knockdown or overexpression cells. *n* = 3. H) Representative images of multiplex fluorescence immunohistochemistry (FDFT1, ALDOB, and DAPI) from HCC tissues with high or low FDFT1 expression. Scale bars, 50 µm. *n* = 5. I) Western blot results show that ALDOB knockdown using siRNA restores AKT1 phosphorylation in FDFT1 knockdown cells. *n* = 3. Data are presented as mean ± SD (C, G‐I). Images were quantified by image J (C and G‐I). Data were analyzed by unpaired t‐test (C, G‐H) or one‐way ANOVA (I) with Bonferroni multiple‐comparison correction. KEGG, Kyoto Encyclopedia of genes and genomes; GSEA, gene set enrichment analysis; DEGs, differentially expressed genes; Co‐IP, coimmunoprecipitation; siRNA, small interfering RNA.

To investigate the mechanism by which FDFT1 promotes HCC progression, we performed RNA sequencing (RNA‐seq) of FDFT1‐knockdown and control cells. We acquired a quantitative RNA expression profile of 60611 items and identified 381 DEGs using a threshold of adjust *p *< 0.05 and | log_2_ (Fold change) | > 1.5 (Figure S5A, Supporting Information). Enrichment analysis was subsequently performed to identify critical functions and pathways associated with these DEGs. Gene Ontology‐biological process (GO‐BP) analysis suggested that these DEGs were involved in regulating cell proliferation, migration, and cholesterol homeostasis (Figure S5B, Supporting Information). Kyoto Encyclopedia of Genes and Genomes (KEGG) pathway analysis revealed significant enrichment of the PI3K‐AKT signaling pathway (**Figure** **4**A). Similarly, GSEA analysis of the external data set also indicated that cholesterol homeostasis, AKT, and mTOR signaling pathways were enriched in the high FDFT1 expression groups (Figure 4B; Figure S5C, Supporting Information). Since AKT activation has been confirmed to be closely related to tumorigenesis in HCC,^[^ ^14^ ^]^ we speculated that FDFT1 regulated AKT phosphorylation in HCC. To test this hypothesis, we first detected phosphorylation of AKT1 at Ser473 and found that FDFT1 knockdown and overexpression decreased and increased AKT1 phosphorylation, respectively (Figure 4C). To further confirm the importance of AKT1 activation in FDFT1 regulating HCC, we used the AKT1 inhibitor (MK‐2206) and agonists (SC79) in FDFT1 overexpression and knockdown cells, respectively.^[^ ^15^ ^]^ The EdU labeling, migration, and invasion assays suggested that the AKT1 agonist restored the inhibitory effects of FDFT1 knockdown on cell proliferation and metastasis (Figure S5D,F, Supporting Information), while the AKT1 inhibitor eliminated the effects of FDFT1 overexpression on promoting cell proliferation and metastasis (Figure S5E,G, Supporting Information).

Considering that AKT1 phosphorylation is regulated by various proteins via protein interaction,^[^ ^16^ ^]^ we next performed coimmunoprecipitation (co‐IP) to detect whether AKT1 phosphorylation is directly regulated by FDFT1. Unfortunately, no interaction between FDFT1 and AKT1 was detected in Huh7 cells (Figure S6A, Supporting Information). This evidence indicated that FDFT1 might indirectly regulate AKT1 phosphorylation. Thus, we hypothesized that FDFT1 influences AKT1 phosphorylation by regulating the expression of other proteins that interact with AKT1. To this end, we used co‐IP and mass spectrometry (MS) analysis to capture proteins that interact with AKT1. 882 proteins were obtained, and 2 molecules (ALDOB and PZP) were common among the DEGs and captured proteins (Figure 4D). Notably, ALDOB can interact with AKT1 and inhibit AKT1 phosphorylation by recruiting PP2A in HCC.^[^ ^17^ ^]^ Our co‐IP results in HCC cells also confirmed that ALDOB can bind to AKT1 (Figure 4E; Figure S6E, Supporting Information). However, we did not detect the interaction between the AKT1 and PZP proteins by the co‐IP assay (Figure S6B, Supporting Information). Meanwhile, in order to avoid false negative results caused by excessively low protein abundance of PZP, we further knocked down the PZP protein using small interfering RNA (PZP is up‐regulated after knocking down FDFT1) and found that PZP knockdown did not recover the down‐regulation of AKT1 phosphorylation induced by FDFT1 knockdown (Figure S6C, Supporting Information). Moreover, we found that FDFT1 expression is negatively associated with ALDOB expression (Figure 4F,G; Figure S6D,F–H, Supporting Information). Importantly, multiplex fluorescence IHC of HCC tissues revealed consistent results (Figure 4H).

To further verify the importance of ALDOB in FDFT1‐mediated regulation of the HCC phenotype and AKT1 activation, we assessed AKT1 phosphorylation after ALDOB knockdown via small interfering RNA (siRNA) in FDFT1‐knockdown cells. As expected, ALDOB knockdown reversed the suppression of AKT1 phosphorylation induced by FDFT1 downregulation (Figure 4I). Proliferation‐related assays revealed that ALDOB knockdown increased proliferation potential and impaired the inhibitory effect of FDFT1 knockdown on HCC cells (Figure S7A,B, Supporting Information). Similarly, ALDOB downregulation attenuated the suppression of metastasis induced by FDFT1 knockdown (Figure S7C,D, Supporting Information). In summary, FDFT11 knockdown weakens AKT1 phosphorylation, and this effect is dependent on ALDOB expression in HCC cells.

### 2.4 FDFT1 Knockdown Promotes ALDOB Expression via Increasing HNF4A Transcriptional Activity

**Figure 5:**
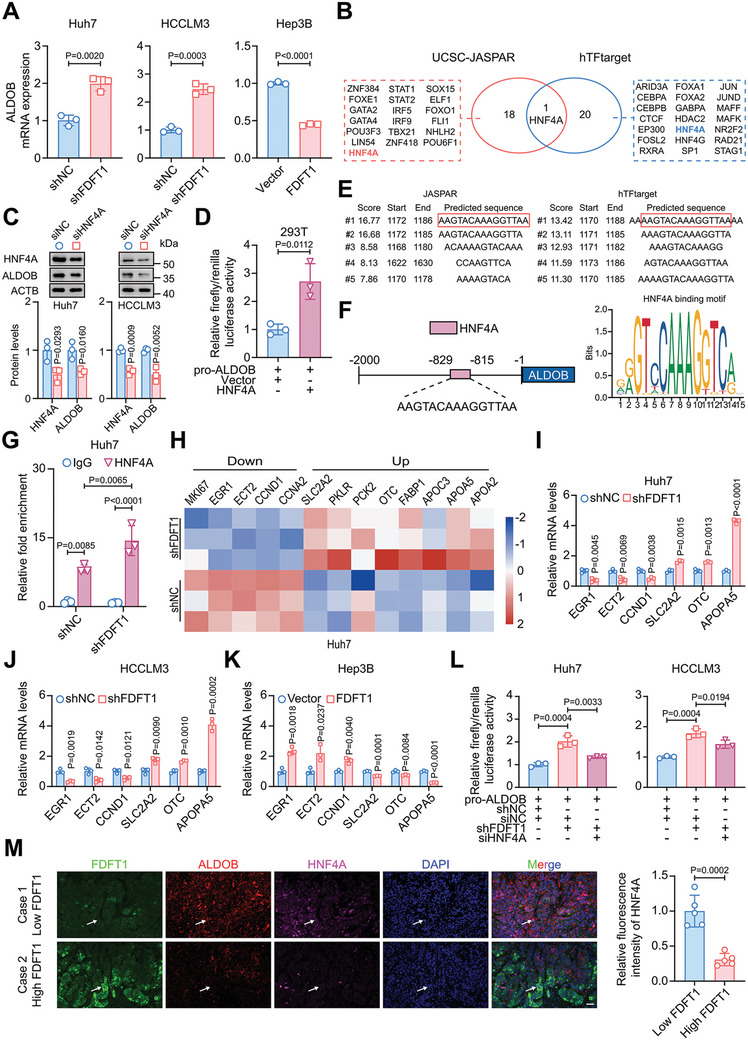
FDFT1 knockdown activates HNF4A to promote ALDOB expression. A) mRNA levels of ALDOB in FDFT1 knockdown or overexpression cells. *n* = 3. B) Venn plot of underlying transcription factors regulating ALDOB in UCSC‐JASPAR and hTFtarget databases. C) Western blot shows HNF4A knockdown decreases ALDOB expression in Huh7 and HCCLM3 cells. *n* = 3. D) Dual‐luciferase reporter assay shows that HNF4A binds to the promoter of ALDOB in 293T cells. *n* = 3. E) Top 5 of underlying biding sites with scores. F) The sequence of underlying biding sites with the highest score (left panel) and HNF4A binding motif (right panel) through the JASPAR database. G) Chip‐qPCR shows HNF4A binds to the promoter of ALDOB via this sequence and FDFT1 knockdown promotes HNF4A activity in Huh7 cells. *n* = 3. H) Hot plot of HNF4A targeted genes in FDFT1 knockdown Huh7 cells. I‐K) mRNA levels of HNF4A targeted genes in FDFT1 knockdown (I‐J) or overexpression (K) cells. *n* = 3. L) Dual‐luciferase reporter assays show that HNF4A knockdown rescues HNF4A activation induced by FDFT1 knockdown. *n* = 3. M) Representative images of multiplex fluorescence immunohistochemistry (FDFT1, ALDOB, HNF4A, and DAPI) from HCC tissues with high or low FDFT1 expression. Scale bars, 50 µm. *n* = 5. Data are presented as mean ± SD (A, C, D, G, I‐M). Images were quantified by image J (C, M). Data were analyzed by unpaired t‐test (A, C, D, I‐K, M), two‐way (G), or one‐way (L) ANOVA with Bonferroni multiple‐comparison correction. pro‐ALDOB, promoter of ALDOB.

Protein levels are regulated mainly by transcription and post‐transcriptional modifications.^[^ ^18^ ^]^ On the basis of our RNA‐seq results, we first speculated that FDFT1 knockdown promotes ALDOB transcription in HCC cells. As expected, ALDOB mRNA levels were elevated and decreased in FDFT1‐knockdown and – overexpression cells, respectively (**Figure** **5**A). To further investigate how FDFT1 regulates ALDOB mRNA levels, we used the University of California Santa Cruz (UCSC) Genome Browser and hTFtarget websites to explore the potential transcription factors that target ALDOB. Notably, only HNF4A was found to be a potential upstream molecule (Figure 5B). Western blotting and qRT‐PCR demonstrated that HNF4A knockdown with siRNAs downregulated ALDOB expression in HCC cells (Figure 5C; Figure S8A, Supporting Information). Importantly, a dual‐luciferase reporter assay in 293T cells confirmed that HNF4A directly binds to the promoter region of *ALDOB* (Figure 5D). Furthermore, we predicted the underlying binding sites and found that the “AAGTACAAAGGTTAA” sequence had the highest score (Figure 5E,F). These results indicate that HNF4A could bind to the promoter region of *ALDOB* and regulate ALDOB expression.

Next, we verified whether HNF4A transcription activity was enhanced after FDFT1 knockdown. We performed chromatin immunoprecipitation (ChIP)‐qPCR based on the “AAGTACAAAGGTTAA” sequence of *ALDOB* and confirmed that HNF4A could bind to this sequence. Importantly, the transcription activity of HNF4A was inhibited after FDFT1 knockdown (Figure 5G). To further validate this finding, we explored the mRNA expression of other HNF4A target genes.^[^ ^19^ ^]^ The sequencing results suggested that FDFT1 knockdown decreased the mRNA levels of downregulated genes (MKI67, EGR1, ECT2, CCND1, and CCNA2) induced by HNF4A, as well as increasing mRNA levels of upregulated genes (SLC2A2, PKLR, PCK2, OTC, FABP1, APOC3, APOA5, and APOA2) induced by HNF4A (Figure 5H). The 6 genes (three upregulated and three downregulated by HNF4A) whose expression levels were most significantly changed in our sequencing data were selected for subsequent experiments. The mRNA expression of these genes was detected via qRT‐PCR, and we obtained consistent results (Figure 5I–K). Additionally, the IHC results confirmed that HNF4A expression was upregulated and downregulated after FDFT1 knockdown and overexpression, respectively (Figure S8B,C, Supporting Information). These results indicate that HNF4A transcription activity was enhanced after FDFT1 was knocked down in HCC cells.

To further explore whether HNF4A plays a crucial role in the regulation of ALDOB and HCC by FDFT1, we altered HNF4A expression in FDFT1‐knockdown or ‐overexpression cells using siRNA or plasmid. Dual‐luciferase reporter and qRT‐PCR assays indicated that HNF4A knockdown abrogated the FDFT1 downregulation‐induced increase in ALDOB transcription, whereas HNF4A overexpression restored ALDOB transcription in FDFT1‐overexpression cells (Figure 5L; Figure S8D–F, Supporting Information). Western blot assays also confirmed these findings (Figure S8G, Supporting Information). In addition, cell proliferation assays indicated that HNF4A antagonized the effect of FDFT1 on the proliferation of HCC cells (Figure S9A–C, Supporting Information). Importantly, multiplex fluorescence immunohistochemistry of HCC tissues demonstrated that HNF4A and ALDOB expression were higher in FDFT1‐low tissues than in FDFT1‐high tissues (Figure 5M). Overall, these findings confirm that ALDOB is the direct target of HNF4A and that FDFT1 knockdown regulates ALDOB and HCC via increasing HNF4A transcriptional activity.

### 2.5 FDFT1 Suppression Reduced Cholesterol and Bile Acid Contents to Enhanced HNF4A Transcriptional Activity

**Figure 6:**
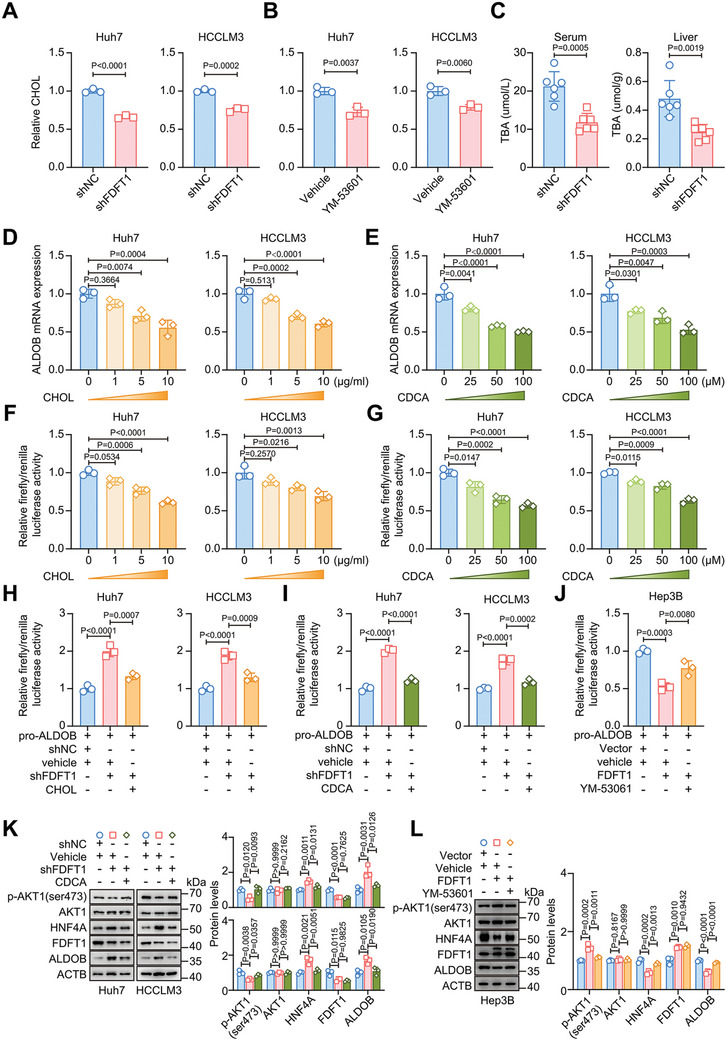
FDFT1 knockdown causes the reduction of cholesterol and bile acid levels to activate HNF4A. A‐B) Relative cholesterol levels in FDFT1 inhibition cells through shRNA (A) or YM‐53061 (an inhibitor of FDFT1 enzyme activity, B). Cells were treated with YM‐53061 (5 µM) for 24 h. *n* = 3. C) Total bile acid levels in serum (left) and liver (right) of HCC mice. *n* = 3. D‐E) The mRNA levels of ALDOB of HCC cells treated with cholesterol (D) or CDCA (E) for 24 h. *n* = 3. F‐G) Dual‐luciferase reporter assays of HCC cells treated with cholesterol (F) or CDCA (G) for 24 h. *n* = 3. H‐I) Dual‐luciferase reporter assays of FDFT1 knockdown cells treated with cholesterol (10 µg mL^−1^, H) or CDCA (100 µM, I) for 24 h. *n* = 3. J) Dual‐luciferase reporter assays of FDFT overexpression cells treated with YM‐53061 (5 µM) for 24 h. *n* = 3. K) Western blot results show AKT1 phosphorylation is recovered in FDFT1 knockdown cells treated with CDCA (100 µM) for 24 h. *n* = 3. L) Western blot results show AKT1 phosphorylation is rescued in FDFT1 overexpression cells treated with YM‐53061 (5 µM) for 24 h. *n* = 3. All data are presented as mean ± SD. Images were quantified by image J (K, L). Data were analyzed by unpaired t‐test (A‐C), and one‐way ANOVA with Bonferroni multiple‐comparison correction (D‐L). CHOL, cholesterol; CDCA, chenodeoxycholic acid.

Previous studies have reported that atorvastatin, a drug widely used to lower cholesterol, elevates HNF4A protein levels^[^ ^20^ ^]^ and that bile acid inhibits the nuclear DNA binding activity of HNF4A in vitro.^[^ ^21^ ^]^ Given the crucial role of FDFT1 in cholesterol synthesis, we hypothesized that FDFT1 knockdown would enhance HNF4A transcriptional activity by reducing cholesterol and bile acid levels. To verify this hypothesis, we first detected cholesterol levels in HCC cells after pharmacologically or genetically inhibiting FDFT1. As expected, FDFT1 inhibition decreased the cholesterol content in HCC cells (**Figure** **6**A,B). Next, we detected total bile acid levels in vivo and found that FDFT1 knockdown caused a reduction in total bile acid levels in the serum and liver (Figure 6C). In addition, transcriptome data from the TCGA cohort suggested that FDFT1 expression was positively associated with the expression of CYP7A1, a major rate‐limiting enzyme involved in bile acid synthesis (Figure S10A, Supporting Information). Moreover, we also found that FDFT1 knockdown and overexpression reduced and increased relative cholesterol and total bile acid levels in xenograft tumors, respectively (Figure. S10B–D, Supporting Information). Notably, cholesterol and chenodeoxycholic acid (CDCA, a primary bile acid) inhibited ALDOB expression and HNF4A transcriptional activity in vitro in a dose‐dependent manner (Figure 6D–G). These results indicated that HNF4A transcriptional activity can be inhibited by cholesterol and bile acid.

In fact, the addition of cholesterol or CDCA reversed the changes in HNF4A transcriptional activity and ALDOB expression induced by FDFT1 knockdown (Figure 6H,I; Figure S10E,F, Supporting Information), whereas the inhibition of FDFT1 enzyme activity by YM‐53081 reactivated HNF4A transcriptional activity (Figure 6J). Moreover, cholesterol or CDCA supplementation restored AKT1 phosphorylation levels in FDFT1‐knockdown cells (Figure 6K; Figure S10G, Supporting Information). In contrast, inhibiting FDFT1 enzyme activity impaired the upregulation of AKT1 phosphorylation induced by FDFT1 overexpression (Figure 6L). Considering the diversity of bile acids, we also examined the effects of other major bile acids on the mRNA levels of ALDOB to verify whether this inhibitory effect is still present. Expectedly, the qRT‐PCR results indicated that the other 4 free bile acids (CA, LCA, DCA, and UDCA) and major glycine (G)/taurine (T)‐conjugated bile acids inhibited the mRNA expression of ALDOB in HCC cells (Figure S10H,I, Supporting Information). In addition, proliferation‐related assays suggested that the inhibition of proliferation potential in FDFT1‐knockdown cells partially depended on cholesterol and bile acid levels (Figure S11A–D, Supporting Information), whereas the promotion of proliferation in FDFT1‐overexpression cells was dependent on FDFT1 enzyme activity (Figure S11E,F, Supporting Information). Collectively, these findings suggest that the cholesterol/bile acid metabolism axis plays a vital role in FDFT1‐mediated regulation of HNF4A transcriptional activity and HCC progression.

### 2.6 Targeting FDFT1 enhances Sensitivity to Capivasertib in HCC

**Figure 7:**
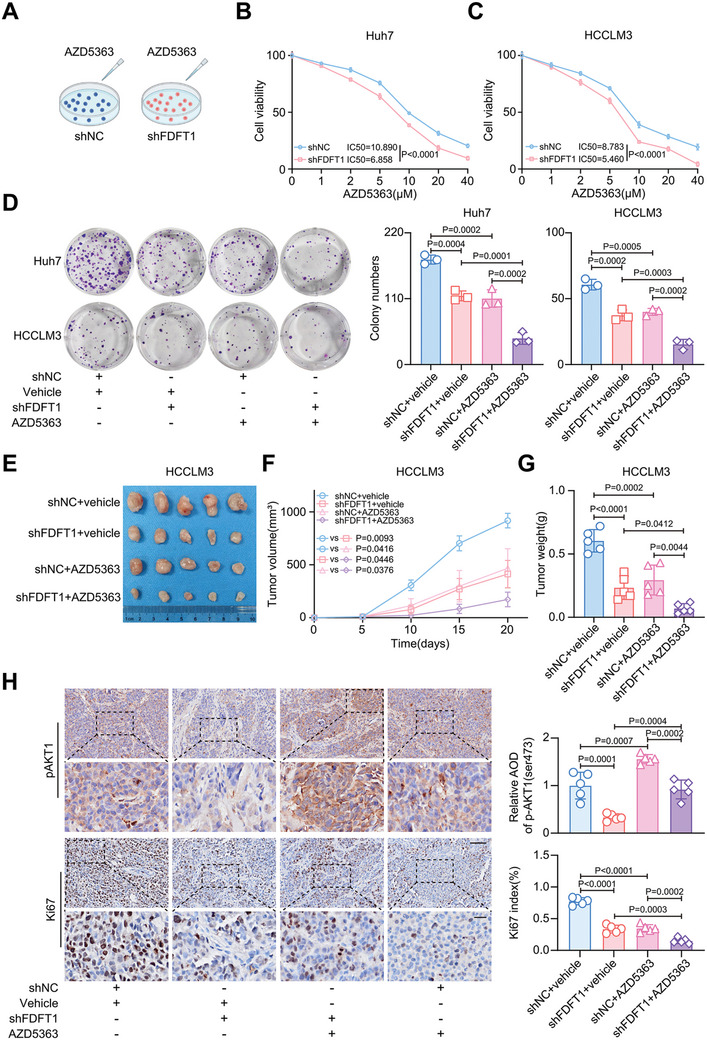
Targeting FDFT1 boosts sensitivity to capivasertib (AZD5363) in HCC. A) Schematic diagram of cells treated with AZD5363 in vitro. B‐C) Cell viability assays of cells treated with AZD5363 at indicated concentrations for 48 h. *n* = 3. D) Representative images of clone formation assays (left panel) and statistical analysis (right panel) of cells with or without FDFT1 knockdown. Cells were treated with AZD5363 (10 µM) for 48 h before cells were implanted into 6‐well plates. *n* = 3. E‐G) Images of tumors harvested at the endpoint (E), tumor volume (F), and tumor weight (G) of subcutaneous xenograft models. Mice were treated with AZD5363 (100 mg k^−1^g, once daily) by oral gavage. *n* = 5. H) Representative images of pAKT1 (ser473) (upper panel) and Ki67 (lower panel) staining of subcutaneous xenograft tumor. Scale bars, 100 (upper panel) and 25 (lower panel) µm. *n* = 5. All data are presented as mean ± SD. Images were quantified by image J (H). Data were analyzed by two‐way (B‐C, F) or one‐way (D, G‐H) ANOVA with Bonferroni multiple‐comparison correction. AOD, average optical density. Image (A) created with BioRender.com. with permission.

Capivasertib (AZD5363), an orally potent inhibitor of AKT kinases, has been proven to have antitumor activity in preclinical studies.^[^ ^22^ ^]^ In HCC, AZD5363 promoted HCC cell apoptosis and augmented the anti‐proliferative efficacy of a dual mTORC1/2 inhibitor.^[^ ^22a^ ^]^ Notably, AZD5363 effectively inhibits the phosphorylation of S6K and 4EBP1.^[^ ^23^ ^]^ However, several studies found that AZD5363 actually increased the phosphorylation level of AKT in several tumor cells.^[^ ^23^ ^]^ In our opinion, we think that as AZD5363 mainly inhibits the downstream targets (S6K and 4EBP1) of AKT, the increased phosphorylation level of AKT may be a compensatory response expression of the cell itself. Considering that a decrease of AKT1 phosphorylation was observed in HCC cells with FDFT1 knockdown, we assessed whether FDFT1 inhibition could impair the compensatory effect. Indeed, FDFT1 knockdown increased the sensitivity of HCC cells to AZD5363 (**Figure** **7**A–D). Importantly, FDFT1 inhibition combined with AZD5363 treatment markedly suppressed the tumor growth in vivo (Figure 7E–G). Consistently. FDFT1 knockdown partially attenuated the increase in AKT1 phosphorylation induced by AZD5363 (Figure 7H). Additionally, Ki67 staining indicated that AZD5363 inhibited tumor proliferation, and this effect was more pronounced when FDFT1 inhibition was combined with AZD5363 treatment (Figure 7H). Moreover, FDFT1 inhibition combined with AZD5363 treatment strongly inhibited phosphorylation of S6K and 4EBP1 (Figure S11A–C, Supporting Information). These results suggest that FDFT1 suppression increases the sensitivity to capivasertib treatment both in vitro and in vivo.

## 3 Discussion

Cholesterol metabolism activation has been observed in a variety of solid tumors, including HCC.^[^ ^24^ ^]^ Statins have some limitations in treating HCC, although statins can be used to lower cholesterol levels. Our study demonstrates that FDFT1 is an important molecule that regulates cholesterol biosynthesis and HCC development. Furthermore, our study indicates that targeting FDFT1 may be a novel potential option for treating HCC.

FDFT1, also known as squalene synthase, determines the conversion of the substrate farnesyl pyrophosphate into squalene in the cholesterol synthesis pathway. Interestingly, the roles of FDFT1 in solid tumors appear to be complex and contradictory. In colorectal and gastric cancers,^[^ ^12a,c^ ^]^ FDFT1 plays a protumoral role, but it plays an anti‐tumoral role in Tongue squamous cell carcinoma and pancreatic cancer.^[^ ^12b,d^ ^]^ Our study systematically reveals that FDFT1 plays a protumoral role in HCC. Although the association between FDFT1 and cholesterol levels has been validated,^[^ ^8^ ^]^ it is unclear whether FDFT1 regulates HCC by affecting cholesterol synthesis. Our work comprehensively confirmed that FDFT1 regulates HCC progression by affecting intracellular cholesterol levels. Intriguingly, cholesterol levels in different microenvironments seem to have the opposite effect on HCC. High cholesterol in the serum enhances the function of nature killer cells and inhibits HCC development,^[^ ^25^ ^]^ whereas cholesterol accumulation in the liver microenvironment impairs nature killer T cells and promotes immune escape.^[^ ^26^ ^]^ Our study focused on cholesterol levels in hepatocytes and confirmed that a reduction in intracellular cholesterol delayed HCC progression. In fact, Yoshinobu Saito et al.^[^ ^8^ ^]^ also reported that the inhibition of cholesterol biosynthesis in hepatocytes suppressed HCC growth via the TAZ/TEAD2 pathway. Bile acids are the main products of cholesterol metabolism in hepatocytes. Our work also verified that bile acids play a protumoral role in HCC, which is consistent with other studies.^[^ ^27^ ^]^ Given the similar commonalities among bile acids, we chose CDCA as the representative bile acid type to conduct relevant experiments.^[^ ^21^, ^27^ ^]^ In fact, the change in cholesterol levels affects the total bile acid pool rather than particular bile acid, so we focus more on changes in the total bile acid pool. Overall, our study contributes to increasing the understanding of the cholesterol in HCC and highlights the importance of bile acids in HCC development.

AKT activation has been widely reported in HCC and served as an important means for the construction of mouse primary liver cancer models.^[^ ^28^ ^]^ Activated AKT phosphorylates PCK1 and then activates SREBP2 for cholesterol synthesis.^[^ ^29^ ^]^ Our findings further reveal cholesterol, upstream of AKT, regulates AKT phosphorylation to promote HCC progression. In fact, previous studies have focused mainly on inducing AKT phosphorylation to increase cholesterol synthesis^[^ ^29^, ^30^ ^]^ while ignoring the effect of intracellular cholesterol on AKT phosphorylation. Our work suggests that there may be a positive feedback loop between cholesterol and AKT phosphorylation. ALDOB, the predominant isoform in the liver, kidney, and small intestine, catalyzes the interconversion of Fructose‐1,6‐bisphosphate (FBP) to dihydroxyacetone phosphate (DHAP) and glyceraldehyde 3‐phosphate (GAP) in glycolysis and gluconeogenesis, respectively.^[^ ^31^ ^]^ Previous studies have revealed that ALDOB is lowly expressed and is an antitumor molecule in HCC.^[^ ^17^, ^31^, ^32^ ^]^ Mechanistically, ALDOB directly binds to AKT and recruits PP2A to promote AKT dephosphorylation.^[^ ^17^ ^]^ Of note, ALDOB can also bind to G6PD to impair the pentose phosphate pathway, leading to HCC suppression.^[^ ^31^ ^]^ Our work further revealed an association between ALDOB and cholesterol metabolism. On the basis of these findings, ALDOB may be a key node linking cholesterol metabolism and glycolysis. Interestingly, cholesterol‐mediated regulation of ALDOB expression is independent of SREBF2 (data not shown). Our work highlights the upstream regulatory mechanism of ALDOB expression in HCC.

HNF4A is a transcription factor that is expressed mainly in hepatocytes.^[^ ^33^ ^]^ In HCC, HNF4A expression is downregulated, leading to HCC aggravation.^[^ ^33^, ^34^ ^]^ However, the molecular mechanisms of HNF4A in HCC need to be further explored. Our study verified that HNF4A can decrease AKT phosphorylation and HCC development via ALDOB. Early studies have suggested that HNF4A regulates ALDOB expression,^[^ ^19e^ ^]^ but whether this regulatory relationship is direct or indirect remains to be further confirmed. Our work verified that HNF4A directly binds to the promoter region of ALDOB to promote ALDOB transcription. Moreover, Zhuo Cheng et al. reported that exogenously expressed HNF4A in HCC cells promotes the conversion of hepatoma cells to hepatocyte‐like cells,^[^ ^35^ ^]^ which strongly supports that HNF4A is important in HCC. Importantly, our work verified that intracellular cholesterol inhibits HNF4A transcription activity via bile acids, providing a novel theoretical basis for targeting cholesterol to treat HCC. Thus far, the molecule mechanisms by which HNF4A inhibits HCC progression still need to be further explored. We revealed that FDFT1 regulates HNF4A transcription activity via the cholesterol/bile acid axis. In fact, Diana Jung et al. also found that the primary bile acid inhibits the nuclear DNA‐binding activity of HNF4A in vitro,^[^ ^21^ ^]^ but they did not assess the HNF4A expression. Moreover, statins promote HNF4A protein expression in aged rats,^[^ ^22a^ ^]^ although it is not known whether this inhibitory effect depends on cholesterol levels. Here, we comprehensively examined the effect of the cholesterol/bile acid axis on HNF4A and found that HNF4A protein expression is inhibited by cholesterol and bile acids. Furthermore, upregulation of HNF4A protein expression induced by the cholesterol/bile acid axis may be associated with the hepatocyte nuclear factor regulatory network.^[^ ^36^ ^]^

Although our work revealed that FDFT1 may be an alternative target against HCC, several shortcomings and unclear mechanisms still need to be further explored. First, we need to strengthen the association between FDFT1 and cholesterol levels in the serum and liver in HCC patients. Second, the upstream regulatory mechanisms of FDFT1 are still unclear and need to be studied. Third, the molecule mechanisms by which HNF4A is regulated by cholesterol and bile acid need to be further explored. Thus, the above limitations will be the focus of our subsequent work. Anyway, our work comprehensively demonstrates the vital role of FDFT1 in cholesterol biosynthesis and HCC and reveals that targeting FDFT1 may be a potential means for treating HCC as an alternative to statins.

## 4 Experimental Section

### Cell Culture and Reagents

Human HCC cell lines (Huh7, Hep3B, SKHep1) were from Cell Bank/Stem Cell Bank, Chinese Academy of Sciences. Human HCC cell lines (HCCLM3) were from the Key Laboratory of Transplant Engineering and Immunology. Human HCC cell lines (Huh7, HCCLM3, SKHep1) were cultured in Dulbecco's modified Eagle's medium (DEME, SH30243.01, Hyclone, USA) while Hep3B was cultured in Minimum Essential Medium (MEM,). All cells were cultured in the complete medium supplemented with 10% fetal bovine serum (FBS, 04‐001‐1A, Biological Industries, Israel) and 1% penicillin/streptomycin (SV30010, Hyclone, USA). All cells were maintained at 37 °C in a 5% CO_2_ atmosphere. All cell lines were analyzed by STR profiling for cell line authentication and routine mycoplasma detection. For reagents, capivasertib (AZD5363), YM‐53601, MK‐2206, SC79 and various indicated bile acids were dissolved with dimethyl sulfoxide and then diluted with indicated medium to make the concentration of dimethyl sulfoxide < 0.1%. Water‐soluble cholesterol was dissolved with ultra‐pure water treated with high temperature and pressure steam sterilization. Capivasertib (HY‐15431), YM‐53601 (HY‐100313A), MK‐2206 (HY‐10358), SC79 (HY‐18749), cholic acid (CA, HY‐N0324), taurocholic acid (TCA, HY‐B1788), glycocholic acid (GCA, HY‐N1423), taurochenodeoxycholic acid (TCDCA, HY‐N2027), glycochenodeoxycholic acid (GCDCA, HY‐N2334), lithocholic acid (LCA, HY‐B0172), taurolithocholic acid (TLCA, HY‐113308), glycolithocholic acid (GLCA, HY‐116374), deoxycholic acid (DCA, HY‐N0593), taurodeoxycholic acid (TDCA, HY‐B1899), glycodeoxycholic acid (GDCA, HY‐125731), ursodeoxycholic acid (UDCA, HY‐13771), tauroursodeoxycholic acid (TUDCA, HY‐19696), glycoursodeoxycholic acid (GUDCA, HY‐N1424) and chenodeoxycholic acid (CDCA, HY‐76847) were purchased from MedChem Express (USA). Cholesterol (c4951) was purchased from Sigma‐Aldrich (USA).

### Clinical Samples Collection

Ten pairs of fresh‐frozen HCC tumors paired adjacent nontumor tissues and 90 paraffin‐embedded HCC tissues were collected for subsequent research. These samples were obtained from West China Hospital of Sichuan University (Chengdu, China) between June 2013 and December 2018. All patients had not received any antitumor therapy before surgery. The clinical diagnosis of HCC was confirmed by pathologic examination of resected specimens. HCC stages were performed on the basis of Barcelona Clinic Liver Cancer (BCLC) staging. Survival time was defined as the interval between the first operation date and the date of death from any cause or the date of the last follow‐up visit. The study involving the use of clinical samples was approved by the Medical Ethics Committee of West China Hospital of Sichuan University and was conducted in accordance with the principles outlined in the Declaration of Helsinki. Informed consent was obtained from all patients or their relatives. The clinical features of the 90 HCC patients are provided in Table S1 (Supporting Information).

### Lentivirus Infection, Small Interfering RNA (siRNA) and Plasmid Transfection

For lentivirus infection, recombination lentiviruses for FDFT1 knockdown (GV344 vector) and overexpression (GV341 vector) were constructed and purchased from GeneChem (China). Small Stable transfection HCC cells were constructed according to the manufacturer's instructions. Cells were then cultured in a complete medium with puromycin (3 µg mL^−1^) for 96 h at 72 h after transfection. Western blot assays were used to detect knockdown and overexpression efficiency. HitransG A regent (REVG004) was purchased from GeneChem (China) and used to assist transfection. For small interfering RNA (siRNA) transfection, siRNAs for *ALDOB, PZP*, and *HNF4A* and the negative control were constructed and purchased from RiboBio (China). Cells were transfected with siRNAs according to the manufacturer's instructions. Cells were analyzed 72 h after transfection. All the reagents used for siRNA transfection were purchased from RiboBio (China). For plasmid transfection, overexpression, and negative control plasmids (GV712 vector) were constructed and purchased from GeneChem (China). According to the manufacturer's instructions, cells were transfected with plasmids using Attractene Transfection Reagent (301004, QIAGEN, Germany). Cells were analyzed 72 h after transfection. The short hairpin RNA (shRNA) and siRNA sequences can be found in Table S2 (Supporting Information).

### Bioinformatics Analysis

The Transcriptomic data of the pan‐GEO dataset from 733 HCC patients was integrated from GSE14520, GSE25097, and GSE36376 through the Gene Expression Omnibus (GEO, https://www.ncbi.nlm.nih.gov/geo/) database. The “sva” package in R was used to remove batch effects and principal component analysis (PCA) was used to evaluate the quality of the integrated data. Similarly, RNA sequence datasets of 365 and 203 HCC patients were collected from the TCGA‐LIHC cohort of The Cancer Genome Atlas (TCGA, https://portal.gdc.cancer.gov/) and ICGC‐LIRI‐JP cohort of the International Cancer Genome Consortium (ICGC, https://dcc.icgc.org/), respectively. HCC samples with incomplete transcriptome data and clinical information were removed. Cholesterol biosynthesis gene sets were obtained from the Molecular Signatures Database (MSigDB, https://www.gsea‐msigdb.org/gsea/msigdb/index.jsp). The gene set enrichment analysis (GSEA) was utilized through the GSEA software (version 4.3.2). Gene Ontology‐biological process (GO‐BP) and Kyoto Encyclopedia of Genes and Genomes (KEGG) analyses were performed by the DAVID database (https://david.ncifcrf.gov/). Differentially expressed genes (DEGs) with adjustive *p* < 0.05 and fold change > 1.5 were obtained by the “limma” package. The best cutoff values of FDFT1 high and low expression grouping in TCGA and ICGC cohorts were determined by “survival” and “survminer” packages.

### Animal Models

Male 4‐week‐old BALB/c nude mice and male and female 3‐month‐old C57BL/6J wild‐type mice for breeding newborn mice were purchased from GemPharmatech (China). All mice were housed in a specific pathogen‐free environment with a 12 h light/dark cycle and controllable light, temperature, and humidity, with food and water available ad libitum. For subcutaneous xenograft models, cells (4 × 10^6^) suspended in 120 µL phosphate‐buffered saline (PBS) were subcutaneously injected into the right flank of nude mice. For drug treatment assays, AZD5363 (100 mg k^−1^g once daily) was dosed by oral gavage in an indicated solvent containing 10% dimethyl sulfoxide, 40% polyethylene glycol 300, 5% tween 80%, and 45% saline. Tumor length and width were recorded at indicated time points and before mice were sacrificed. Tumor volume was calculated as follows: volume (mm^3^) = length × (width)^2^ × 0.52. Indicated days later, mice were sacrificed and tumors were isolated and weighed. For lung metastasis models, cells (1.5 × 10^6^) were injected into the tail vein of nude mice. 2 months later, tumor formation and metastasis were visualized by IVIS@ Lumina II system (Caliper Life Sciences) after intraperitoneal injection of D‐luciferin (150 mg k^−1^g). Then, mice were sacrificed at the indicated times, and lung tissues were excised, fixed in 4% formaldehyde, and embedded in paraffin for further detection. For primary liver cancer models, primary liver cancer was induced by treatment with diethylnitrosamine (DEN) and carbon tetrachloride (CCl_4_). Male 1‐week‐old wild‐type mice were injected adeno‐associated virus type 8 (AAV8, 2 × 10^11^ vg mice^−1^, Vigene, China) with liver‐specific promoter thyroxine‐binding globulin (TBG) into the tail vein. Male 2‐week‐old wild‐type mice were intraperitoneally injected with DEN (25 mg k^−1^g). At the age of 4 weeks, mice were intraperitoneally injected with 10% CCl_4_ (5 mL kg^−1^) every week till sacrificed. Liver and serum were collected for further analysis. IHC of liver tissues was used to evaluate the efficiency of FDFT1 knockdown. Targeting FDFT1 and control sequences can be found in Table S2 (Supporting Information).

### Western Blot

Tissue and cell samples were homogenized in RIPA buffer (P0013B, Beyotime, China) with proteinase inhibitor and phosphatase inhibitor (M5293/M7528, Abmole, USA). All operations were performed according to the manufacturer's instructions. Briefly, protein supernatant was collected and protein concentration was determined using the BCA assay kit (P0012S, Beyotime, China). Equal amounts of the protein from the supernatant were separated in 10% sodium dodecyl sulfate (SDS) polyacrylamide gels (ZD304A‐2, Zoman, China) and transferred onto polyvinylidene difluoride (PVDF) membrane (ISEQ00005, Millipore, USA), and then blotted with antibodies. The immunoblots were visualized by chemiluminescence (4AW011‐100, 4abio, China). The primary and second antibodies used in this study are listed in Table S3 (Supporting Information).

### Immunofluorescence

Cells were implanted into 24‐well plates and grown on coverslips. When the cell fusion was ≈70%, cells were washed with PBS and then fixed with 4% paraformaldehyde solution for 20 min. After being drilled with 0.1% Triton X‐100 for 20 min, samples were blocked with 5% BSA for 1 h at room temperature and then incubated with primary antibodies overnight at 4 °C. Next day, samples were incubated with corresponding secondary antibodies for 1 h at room temperature and viewed using a fluorescence microscope (Olympus) at the same exposure. Antibodies used in the section are listed in Table S3 (Supporting Information).

### Immunohistochemistry (IHC) and Multiplex Fluorescence Immunohistochemistry

Paraffin‐embedded tissues from humans or mice were used. Briefly, sections were first incubated in 3% H_2_O_2_ for 30 min at 37 °C after dewaxing. Next, sections were processed for antigen retrieval in a microwave and incubated in Ethylene Diamine Tetraacetic Acid (EDTA) buffer for 20 min. Then, sections were blocked with endogenous peroxidase blockers (PV‐9001, ZSGB‐BIO, China). Indicated proteins were detected through specific primary antibodies and the horseradish peroxidase (HRP)‐conjugated secondary antibody. Multiplex fluorescence immunohistochemistry was performed using a Pika three‐label four‐color fluorescence detection kit (RS0035‐1, ImmunoWay, USA). Briefly, sections were incubated with primary antibodies for 4 h at room temperature after antigen retrieval and blocking. Next, sections were incubated with secondary antibodies for 1 h and then incubated with corresponding fluorochrome for 1 h at room temperature. Then, the above steps were repeated according to the experimental requirements. Sections were viewed using a fluorescence microscope (Olympus) at the same exposure. Antibodies used in the section are listed in Table S3 (Supporting Information).

### Cell counting Kit‐8 (CCK8) Assay

The CCK8 assay was performed through CCK8 kit (M4839, Abmole, USA) according to the manufacturer's instructions. Briefly, cells (3000–5000 per well) were inoculated in 96‐well plates and cultured with the indicated medium. Before detection, 10 µL of CCK8 solution was added to the wells and incubated for 2 h. The absorbance at 450 nm was detected at the indicated time points. Half maximal inhibitory concentration (IC50) was calculated after 48 h of drug action through GraphPad Prism (version 9.3.1).

### Colony Formation Assay

Cells (500 per well) were implanted into 6‐well plates and cultured with a complete medium for up to 14 days. Cells were then immobilized with 4% paraformaldehyde solution and stained with 0.1% crystal violet staining solution. ImageJ was used for clone count.

### EdU (5‐ethynyl‐2′‐deoxyuridine) Labelling Assay

Edu staining assay was performed through an EdU kit (C10310‐1, RiboBio, China) according to the manufacturer's instructions. Briefly, cells were grown on coverslips and incubated with the indicated medium containing EdU. After being fixed and drilled, cells were stained through the Apollo and 4′,6‐diamidino‐2‐phenylindole (DAPI, 28718‐90‐3, Sigma‐Aldrich, USA) staining solutions. Cells were finally viewed using a fluorescence microscope (Olympus) at the same exposure. ImageJ was used for EdU positive rate statistics of cells.

### Transwell Assays

Cells (2–5 × 10^4^ per well) resuspended in FBS‐free medium were added to the upper chamber (8.0 µm pore size) coated without (migration) or with (invasion) 2atrigel. The bottom chamber was filled with 10% FBS as an inducer and cells in the upper chamber were cultured in 24‐well plates for 24 to 48 h. Later, the cells that migrated to the bottom surface of the chamber were fixed with 4% paraformaldehyde, stained with 0.1% crystal violet, captured by microscope, and counted by ImageJ.

### Serum Alanine Transaminase (ALT), Aspartate Aminotransferase (AST), Total Cholesterol and Bile Acid Detection

ALT, AST, total cholesterol, and bile acids levels in serum were detected by the 126 laboratories from West China Hospital of Sichuan University.

### Cholesterol and Total Bile Acid Detection

Cholesterol contents in cells and liver tissues were determined by the total cholesterol assay kit (A111‐1‐1, Nanjing Jiancheng Bioengineering Institute, China) according to the manufacturer's instructions. The absorbance at 500 nm was detected. Cholesterol contents were calculated according to the manufacturer's instructions and relative contents were normalized by the control group. Total bile acid contents in liver tissues were determined by the total bile acid assay kit (E‐BC‐K181‐M, Elabscience, China) according to the manufacturer's instructions. The absorbance at 405 nm was detected. Total bile acid contents were calculated according to the manufacturer's instructions.

### RNA Sequencing (RNA‐seq) and Data Analysis

Total RNA from Huh7 cells was isolated using Trizol reagent (15596026CN, Thermo fisher Scientific, USA). The total RNA samples were then sent to Lc‐Bio Technologies (China) for subsequent library construction and absolute quantitative transcriptome sequencing. Briefly, the eukaryotic mRNA was enriched using magnetic beads connected with Oligo (dT) after the total RNA was qualified, and then the mRNA was randomly broken into short fragments. Using the fragment mRNA as a template, a cDNA strand was synthesized with six‐base random primers, and then the adapter with UMI was connected. Finally, PCR amplification was performed to obtain the final sequencing library. The library was sequenced by Illumina Novaseq 6000. After completing the sequencing, reads of samples was aligned to the UCSC (http://genome.ucsc.edu/) homo sapiens reference genome using the HISAT package.^[^ ^37^ ^]^ The mapped reads of each sample were assembled using StringTie^[^ ^38^ ^]^ and all transcriptomes from Samples were merged to reconstruct a comprehensive transcriptome. Then, StringTie was used to perform expression levels for mRNAs by calculating fragments per kilobase of exon model per million mapped fragments (FPKM). The differentially expressed genes (DEGs) were selected with log2 (fold change) > 1.5 or log2 (fold change) < −1.5 and with statistical significance (adjust *p* value < 0.05) by edgeR package.

### Coimmunoprecipitation (CoIP) and Mass Spectrometry (MS)

CoIP assays were performed using a coimmunoprecipitation kit (abs955, absin, China). 500 uL cell lysate was prepared according to instructions and added to 5 uL protein A and G for pre‐cleaning. After centrifugation, the liquid supernatant was incubated overnight at 4 °C with 5 uL protein A and G agarose beads and 5ug indicated antibody. Then, the beads were washed 3 times with wash buffer and immunoprecipitates were collected through centrifugation. Finally, immunoprecipitates were resuspended with 40ul SDS loading buffer and analyzed by western blot. For mass spectrometry, samples were processed and identified by Oebioteche (China). Briefly, the samples were tested by Nano‐HPLC Liquid Phase System UltiMate 3000 RSLCnano (ThermoFisher Scientific, USA) after enzymatic hydrolysis and desalting. Then, the raw protein file was directly imported into the Proteome Discoverer software (version 2.5) for subsequent analysis. The antibodies involved in this section are listed in Table S3 (Supporting Information).

### Real‐Time Quantitative Reverse Transcription PCR (qRT‐PCR)

Total RNAs were isolated using a Cell Total RNA Isolation Kit (RE‐03111, Foregene, China). Next, 1ug total RNA was synthesized into cDNA by iScript cDNA Synthesis Kit (1 708 890, Bio‐Rad, USA). Then, real‐time PCR was performed using QuantiNova SYBR Green PCR Kit (208 054, QIAGEN, Germany). The relative expression of indicated RNA was calculated by the 2^−ΔΔCt^ method and normalized by GAPDH. All processing was performed according to the instructions. Primers used in this section are synthesis by Sangon Biotech (China) and shown in Table S4 (Supporting Information).

### Transcription Factor Analysis

The underlying promoter sequence was determined by 2000bp upstream and acquired through the National Center for Biotechnology Information (NCBI, https://www.ncbi.nlm.nih.gov/). Next, candidate transcription factors were predicted through the JASPAR 2022 tool (track score ≥ 500) of the University of California Santa Cruz (UCSC) Genome Browser (http://www.genome.ucsc.edu/) and hTFtarget (https://guolab.wchsc‐u.cn/hTFtarget//#!/). Then, predicted site sequences were obtained through JASPAR (https://jaspar.elixir.no/) and hTFtarget.

### Dual‐Luciferase Reporter Assay

The luciferase reporter plasmids (GV238 vector) containing the ALDOB promoter sequence were constructed by GeneChem (China). Cells were seeded in 24‐well plates for 24 h and co‐transfected with GV238 and Renilla luciferase plasmids, with or without HNF4A overexpression plasmids using Attractene Transfection Reagent (301 004, QIAGEN, Germany). Luciferase activity was measured 72 h post‐transfection using the Dual‐Luciferase Reporter Gene Assay Kit II (RG029S, Beyotime, China). Luciferase activity value was normalized by the Renilla activity value and the ratio was then normalized by the control ratio.

### Chromatin Immunoprecipitation (ChIP)‐qPCR

ChIP assays were performed according to the manufacturer's instructions. Briefly, indicated cells were fixed with 1% formaldehyde for 10 min. The supernatant containing digested chromatin was acquired after lysis and MNase digestion and centrifugation, and the 10% total sample was the input group. Then, chromatin lysates were incubated with indicated antibodies or normal rabbit IgG overnight at 4 °C, and immunoprecipitated chromatin was captured with ChIP Grade Protein A/G Plus Agarose. Finally, purified DNA was obtained through elution and DNA recovery, and assessed by quantitative real‐time PCR (qPCR) using QuantiNova SYBR Green PCR Kit (208 054, QIAGEN, Germany). Primers of ChIP‐qPCR were designed by Primer‐BLAST (https://blast.ncbi.nlm.nih.gov/Blast.cgi) and synthesis by Sangon Biotech (China). Relative fold enrichment was normalized by control rabbit IgG. Antibodies and the primer sequences used in this section are listed in Tables S3 and S4 (Supporting Information), respectively.

### Statistical Analysis

All the statistical analyses in this study were performed using GraphPad Prism (version 9.3.1) or R software (version 4.3.1). All the statistical data were presented as the mean ± standard deviations (SDs). Clinical characteristics were compared via a chi‐square test. Other data were analyzed via two‐tailed Student's t‐test or ANOVA. The detailed statistical methods used for each experiment can be found in the figure legends. Linear correlations were analyzed via Spearman correlation analysis. *p *< 0.05 was considered to indicate statistical significance.

### Ethical Statement

The study involving the use of clinical samples was approved by the Medical Ethics Committee of West China Hospital of Sichuan University and was conducted in accordance with the principles outlined in the Declaration of Helsinki. Informed consent was obtained from all patients or their relatives. The animal care and experimental procedures were conducted in accordance with the Guide for the Care and Use of Laboratory Animals and were approved by the Animal Care and Use Committee of Sichuan University.

## Conflict of Interest

The authors have no conflicts to report.

## Author Contributions

D.C., G.‐C.Z., and X.D. contributed equally to this work and shared the first authorship. D.C., G.‐C.Z., and X.D. conceived the project and designed the research. The order of the co‐first authors was assigned based on their intellectual and experimental contributions. D.C., G.‐C.Z., D.X., Z.Z., M.C., J.H., and Z.W. performed experiments. D.C., G.‐C.Z., X.D., Z.Z., M.C., J.H., Z.W., and L.C. analyzed the data. M.C. and Z.W. provided clinical samples. D.C., G.‐C.Z., and X.D. drafted and revised the manuscript. G.‐C.Z., Z.Z., M.C., J.H., Z.W., L.C., S.L., and J.G. revised the manuscript. S.L. and J.G. conceived and oversaw the research. All the authors approved the final version of the manuscript.

## Supporting information



Supporting Information

## Data Availability

The data that support the findings of this study are available from the corresponding author upon reasonable request.

## References

[1] F. Bray , M. Laversanne , H. Sung , J. Ferlay , R. L. Siegel , I. Soerjomataram , A. Jemal , CA Cancer J. Clin. 2024, 74, 229. 38572751 10.3322/caac.21834

[2] J. M. Llovet , R. K. Kelley , A. Villanueva , A. G. Singal , E. Pikarsky , S. Roayaie , R. Lencioni , K. Koike , J. Z. Rossi , Nat. Rev. Dis. Primers. 2021, 7, 6. 33479224 10.1038/s41572-020-00240-3 PMC13537433

[3] a) L. Viganò , S. Conci , M. Cescon , C. Fava , P. Capelli , A. D'Errico , G. Torzilli , L. D. Tommaso , F. Giuliante , F. M. Vecchio , M. Salizzoni , E. David , A. D. Pinna , A. Guglielmi , L. Capussotti , J. Hepatol. 2015, 63, 93; 25646890 10.1016/j.jhep.2015.01.024

[4] a) S. Qin , M. Kudo , T. Meyer , Y. Bai , Y. Guo , Z. Meng , T. Satoh , D. Marino , E. Assenat , S. Li , Y. Chen , F. Boisserie , R. Abdrashitov , R. S. Finn , A. Vogel , A. X. Zhu , JAMA Oncol. 2023, 9, 1651; 37796513 10.1001/jamaoncol.2023.4003 PMC10557031

[5] H. Li , X. H. Yu , X. Ou , X. P. Ouyang , C. K. Tang , Prog. Lipid Res. 2021, 83, 101109. 34097928 10.1016/j.plipres.2021.101109

[6] D. Hanahan , R. A. Weinberg , Cell. 2011, 144, 646. 21376230 10.1016/j.cell.2011.02.013

[7] a) Q. Gao , H. Zhu , L. Dong , W. Shi , R. Chen , Z. Song , C. Huang , J. Li , X. Dong , Y. Zhou , Q. Liu , L. Ma , X. Wang , J. Zhou , Y. Liu , E. Boja , A. I. Robles , W. Ma , P. Wang , Y. Li , L. Ding , B. Wen , B. Zhang , H. Rodriguez , D. Gao , H. Zhou , J. Fan , Cell. 2019, 179, 561; 31585088 10.1016/j.cell.2019.08.052

[8] Y. Saito , D. Yin , N. Kubota , X. Wang , A. Filliol , H. Remotti , A. Nair , L. Fazlollahi , Y. Hoshida , I. Tabas , K. J. Wangensteen , R. F. Schwabe , Gastroenterology. 2023, 164, 1279. 36894036 10.1053/j.gastro.2023.02.043 PMC10335360

[9] A. Oesterle , U. Laufs , J. K. Liao , Circ. Res. 2017, 120, 229. 28057795 10.1161/CIRCRESAHA.116.308537 PMC5467317

[10] a) J. L. Jouve , T. Lecomte , O. Bouché , E. Barbier , F. K. Akouz , G. Riachi , E. N. Khac , I. Ollivier‐Hourmand , M. Debette‐Gratien , R. Faroux , A. L. Villing , J. Vergniol , J. F. Ramee , J. P. Bronowicki , J. F. Seitz , J. L. Legoux , J. Denis , S. Manfredi , J. M. Phelip , J. Hepatol. 2019, 71, 516; 31125576 10.1016/j.jhep.2019.04.021

[11] E. Pose , J. Trebicka , R. P. Mookerjee , P. Angeli , P. Ginès , J Hepatol. 2019, 70, 194. 30075229 10.1016/j.jhep.2018.07.019

[12] a) M. Weng , W. Chen , X. Chen , H. Lu , Z. Sun , Q. Yu , P. Sun , Y. Xu , M. Zhu , N. Jiang , J. Zhang , J. Zhang , Y. Song , D. Ma , X. Zhang , C. Miao , Nat. Commun. 2020, 11, 1869; 32313017 10.1038/s41467-020-15795-8 PMC7170903

[13] G. G. Jinesh , A. S. Brohl , Signal Transduct. Target Ther. 2022, 7, 296. 35999218 10.1038/s41392-022-01132-6 PMC9399134

[14] Y. Wang , K. Lin , S. Chen , D. Gu , C. Chen , P. Tu , Y. Jou , Hepatology. 2013, 58, 239. 23460382 10.1002/hep.26352

[15] a) N. Grabinski , F. Ewald , B. Hofmann , K. Staufer , U. Schumacher , B. Nashan , M. Jücker , Mol. Cancer. 2012, 11, 85; 23167739 10.1186/1476-4598-11-85 PMC3545733

[16] Y. He , M. Sun , G. Zhang , J. Yang , K. Chen , W. Xu , B. Li , Signal Transduct. Target Ther. 2021, 6, 425. 34916492 10.1038/s41392-021-00828-5 PMC8677728

[17] a) X. He , M. Li , H. Yu , G. Liu , N. Wang , C. Yin , Q. Tu , G. Narla , Y. Tao , S. Cheng , H. Yin , PLoS Biol. 2020, 18, e3000803; 33275593 10.1371/journal.pbio.3000803 PMC7744066

[18] a) Y. Geffen , S. Anand , Y. Akiyama , T. M. Yaron , Y. Song , J. L. Johnson , A. Govindan , Ö. Babur , Y. Li , E. Huntsman , L. Wang , C. Birger , D. I. Heiman , Q. Zhang , M. Miller , Y. E. Maruvka , N. J. Haradhvala , A. Calinawan , S. Belkin , A. Kerelsky , K. R. Clauser , K. Krug , S. Satpathy , S. H. Payne , D. R. Mani , M. A. Gillette , S. M. Dhanasekaran , M. Thiagarajan , M. Mesri , H. Rodriguez , et al., Cell. 2023, 186, 3945; 37582358 10.1016/j.cell.2023.07.013 PMC10680287

[19] a) C. P. Martínez‐Jiménez , M. J. Gómez‐Lechón , J. V. Castell , R. Jover , J. Biol. Chem. 2006, 281, 29840; 16891307 10.1074/jbc.M604046200

[20] E. Sanguino , N. Roglans , M. Alegret , R. M. Sánchez , M. Vázquez‐Carrera , J. C. Laguna , Br. J. Pharmacol. 2005, 145, 853. 15912134 10.1038/sj.bjp.0706260 PMC1576214

[21] D. Jung , G. A. Kullak‐Ublick , Hepatology. 2003, 37, 622. 12601360 10.1053/jhep.2003.50100

[22] a) T. Patra , K. Meyer , R. B. Ray , T. Kanda , R. Ray , Cell Death Dis. 2021, 12, 1073; 34759291 10.1038/s41419-021-04371-7 PMC8580964

[23] a) L. Lang , C. Shay , X. Zhao , Y. Xiong , X. Wang , Y. Teng , J. Hematol. Oncol. 2019, 12, 132; 31805962 10.1186/s13045-019-0827-1 PMC6896687

[24] F. Bovenga , C. Sabbà , A. Moschetta , Cell Metab. 2015, 21, 517. 25863245 10.1016/j.cmet.2015.03.002

[25] W. Qin , Z. Yang , M. Li , Y. Chen , X. Zhao , Y. Qin , J. Song , B. Wang , B. Yuan , X. Cui , F. Shen , J. He , Y. Bi , G. Ning , J. Fu , H. Wang , Gastroenterology. 2020, 158, 1713. 31972238 10.1053/j.gastro.2020.01.028

[26] W. Tang , J. Zhou , W. Yang , Y. Feng , H. Wu , M. T. S. Mok , L. Zhang , Z. Liang , X. Liu , Z. Xiong , X. Zeng , J. Wang , J. Lu , J. Li , H. Sun , X. Tian , P. C. Yeung , Y. Hou , H. M. Lee , C. C. H. Lam , H. H. W. Leung , A. W. H. Chan , K. F. To , J. Wong , P. B. S. Lai , K. K. C. Ng , S. K. H. Wong , V. W. S. Wong , A. P. S. Kong , J. J. Y. Sung , et al., Cell Mol. Immunol. 2022, 19, 834. 35595819 10.1038/s41423-022-00872-3 PMC9243114

[27] a) R. Sun , Z. Zhang , R. Bao , X. Guo , Y. Gu , W. Yang , J. Wei , X. Chen , L. Tong , J. Meng , C. Zhong , C. Zhang , J. Zhang , Y. Sun , C. Ling , X. Tong , F. Yu , H. Yu , W. Qu , B. Zhao , W. Guo , M. Qian , H. Saiyin , Y. Liu , R. Liu , C. Xie , W. Liu , Y. Xiong , K. Guan , Y. Shi , et al., J. Hepatol. 2022, 77, 453; 35292350 10.1016/j.jhep.2022.02.030

[28] J. Sun , J. Ding , Q. Shen , X. Wang , M. Wang , Y. Huang , X. Zhang , H. Zhu , F. Zhang , D. Wu , M. Peng , Z. Zhang , Y. Yuan , W. Li , Z. She , X. Zhang , H. Li , P. Zhang , Z. Huang , J. Hepatol. 2023, 78, 627. 36462680 10.1016/j.jhep.2022.11.017

[29] D. Xu , Z. Wang , Y. Xia , F. Shao , W. Xia , Y. Wei , X. Li , X. Qian , J. Lee , L. Du , Y. Zheng , G. Lv , J. Leu , H. Wang , D. Xing , T. Liang , M. Hung , Z. Lu , Nature. 2020, 580, 530. 32322062 10.1038/s41586-020-2183-2

[30] D. Liu , C. C. Wong , L. Fu , H. Chen , L. Zhao , C. Li , Y. Zhou , Y. Zhang , W. Xu , Y. Yang , B. Wu , G. Cheng , P. B‐S. Lai , N. Wong , J. J. Y. Sung , J. Yu , Sci. Transl. Med. 2018, 10, eaap9840. 29669855 10.1126/scitranslmed.aap9840

[31] M. Li , X. He , W. Guo , H. Yu , S. Zhang , N. Wang , G. Liu , R. Sa , X. Shen , Y. Jiang , Y. Tang , Y. Zhuo , C. Yin , Q. Tu , N. Li , X. Nie , Y. Li , Z. Hu , H. Zhu , J. Ding , Z. Li , T. Liu , F. Zhang , H. Zhou , S. Li , J. Yue , Z. Yan , S. Cheng , Y. Tao , H. Yin , Nat. Cancer. 2020, 1, 735. 35122041 10.1038/s43018-020-0086-7

[32] Q. Tao , S. Yuan , F. Yang , S. Yang , Y. Yang , J. Yuan , Z. Wang , Q. Xu , K. Lin , J. Cai , J. Yu , W. Huang , X. Teng , C. Zhou , F. Wang , S. Sun , W. Zhou , Mol. Cancer. 2015, 14, 170. 26376879 10.1186/s12943-015-0437-7 PMC4574028

[33] B. Ning , J. Ding , C. Yin , W. Zhong , K. Wu , X. Zeng , W. Yang , Y. Chen , J. Zhang , X. Zhang , H. Wang , W. Xie , Cancer Res. 2010, 70, 7640. 20876809 10.1158/0008-5472.CAN-10-0824

[34] Q. Xu , Y. Li , X. Gao , K. Kang , J. G. Williams , L. Tong , J. Liu , M. Ji , L. J. Deterding , X. Tong , J. W. Locasale , L. Li , I. Shats , X. Li , Nat. Commun. 2020, 11, 3978. 32770044 10.1038/s41467-020-17818-w PMC7414133

[35] Z. Cheng , Z. He , Y. Cai , C. Zhang , G. Fu , H. Li , W. Sun , C. Liu , X. Cui , B. Ning , D. Xiang , T. Zhou , X. Li , W. Xie , H. Wang , J. Ding , Cell Res. 2019, 29, 124. 30560924 10.1038/s41422-018-0111-x PMC6355772

[36] D. T. Odom , N. Zizlsperger , D. B. Gordon , G. W. Bell , N. J. Rinaldi , H. L. Murray , T. L. Volkert , J. Schreiber , P. A. Rolfe , D. K. Gifford , E. Fraenkel , G. I. Bell , R. A. Young , Science. 2004, 303, 1378. 14988562 10.1126/science.1089769 PMC3012624

[37] D. Kim , B. Langmead , S. L. Salzberg , Nat. Methods. 2015, 12, 357. 25751142 10.1038/nmeth.3317 PMC4655817

[38] M. Pertea , G. M. Pertea , C. M. Antonescu , T. Chang , J. T. Mendell , S. L. Salzberg , Nat. Biotechnol. 2015, 33, 290. 25690850 10.1038/nbt.3122 PMC4643835

