# Supplementary material for: Targeting FDFT1 Reduces Cholesterol and Bile Acid Production and Delays Hepatocellular Carcinoma Progression Through the HNF4A/ALDOB/AKT1 Axis
Source: Adv Sci (Weinh). 2025 Feb 3;12(12):2411719. doi: 10.1002/advs.202411719 (PMC11948044; doi:10.1002/advs.202411719)
Supplement: Supplementary file 1 — Supporting Information [file ADVS-12-2411719-s001.docx]

Supporting Information

**Targeting FDFT1 reduces cholesterol and bile acid production and delays hepatocellular carcinoma progression through the HNF4A/ALDOB/AKT1 axis**

*Dong Cai, Guo-chao Zhong, Xin Dai, Zhibo Zhao, Menglin Chen, Jiejun Hu, Zhenru Wu, Lve Cheng, Shengwei Li,* and Jianping Gong**


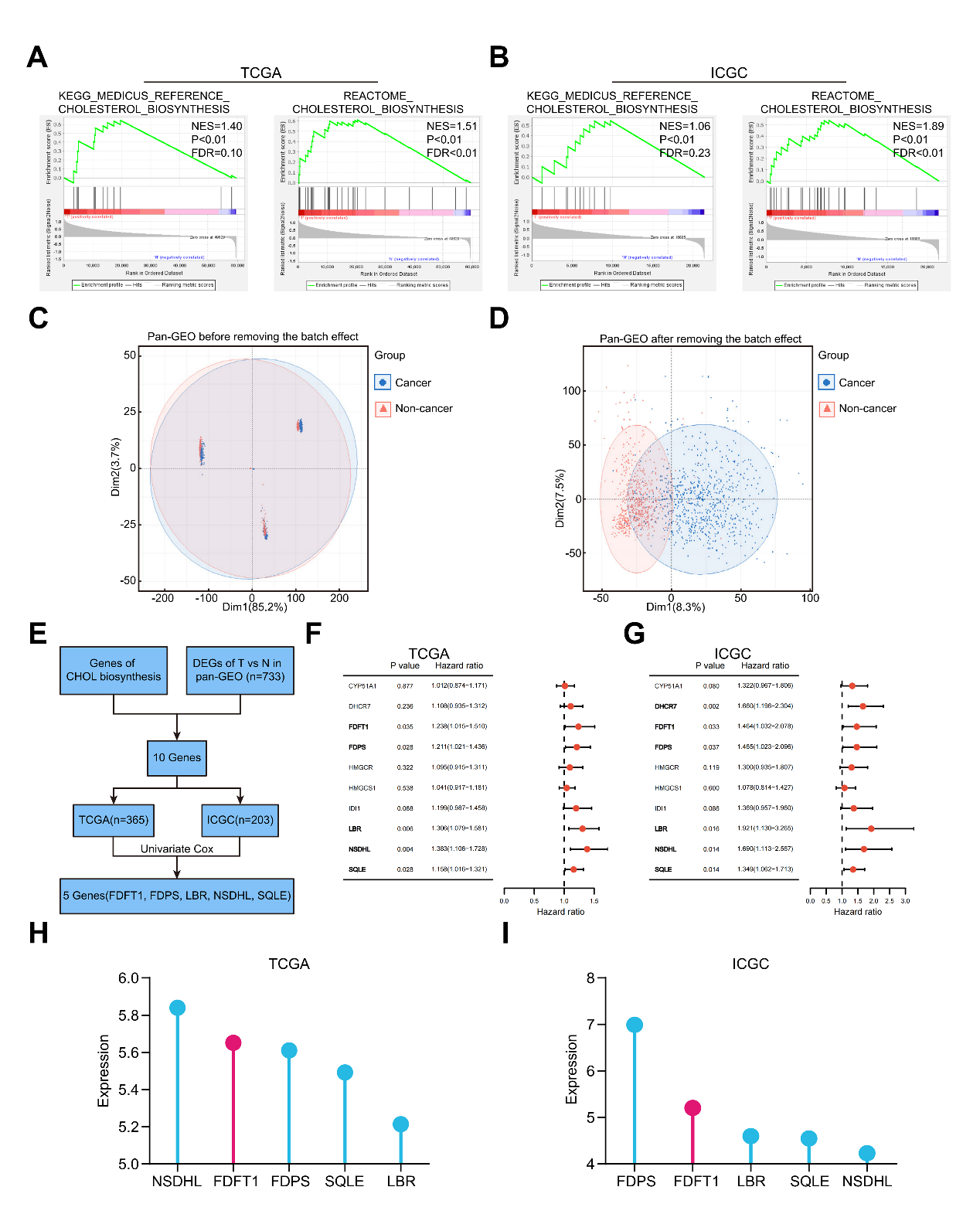
Figure S1. Cholesterol biosynthesis is increased in HCC and identifying FDFT1 as the potential key target. A-B) GSEA analysis of cholesterol biosynthesis (gene sets from KEGG and reactome) in HCC patients from TCGA (A) and ICGC (B) cohorts. C-D) PCA plots of the integrated GEO data set (GSE14520, GSE25097 and GSE36376) before (C) and after (D) batch correction. E) Flow chart of screening 5 genes (FDFT1, FDPS, LBR, NSDHL and SQLE). F-G) Univariate Cox regression analysis of 11 genes in TCGA (F) or ICGC (G) cohorts. H-I) Expression of 5 genes in HCC patients from TCGA (H) or ICGC (I) cohorts. GSEA, gene set enrichment analysis.


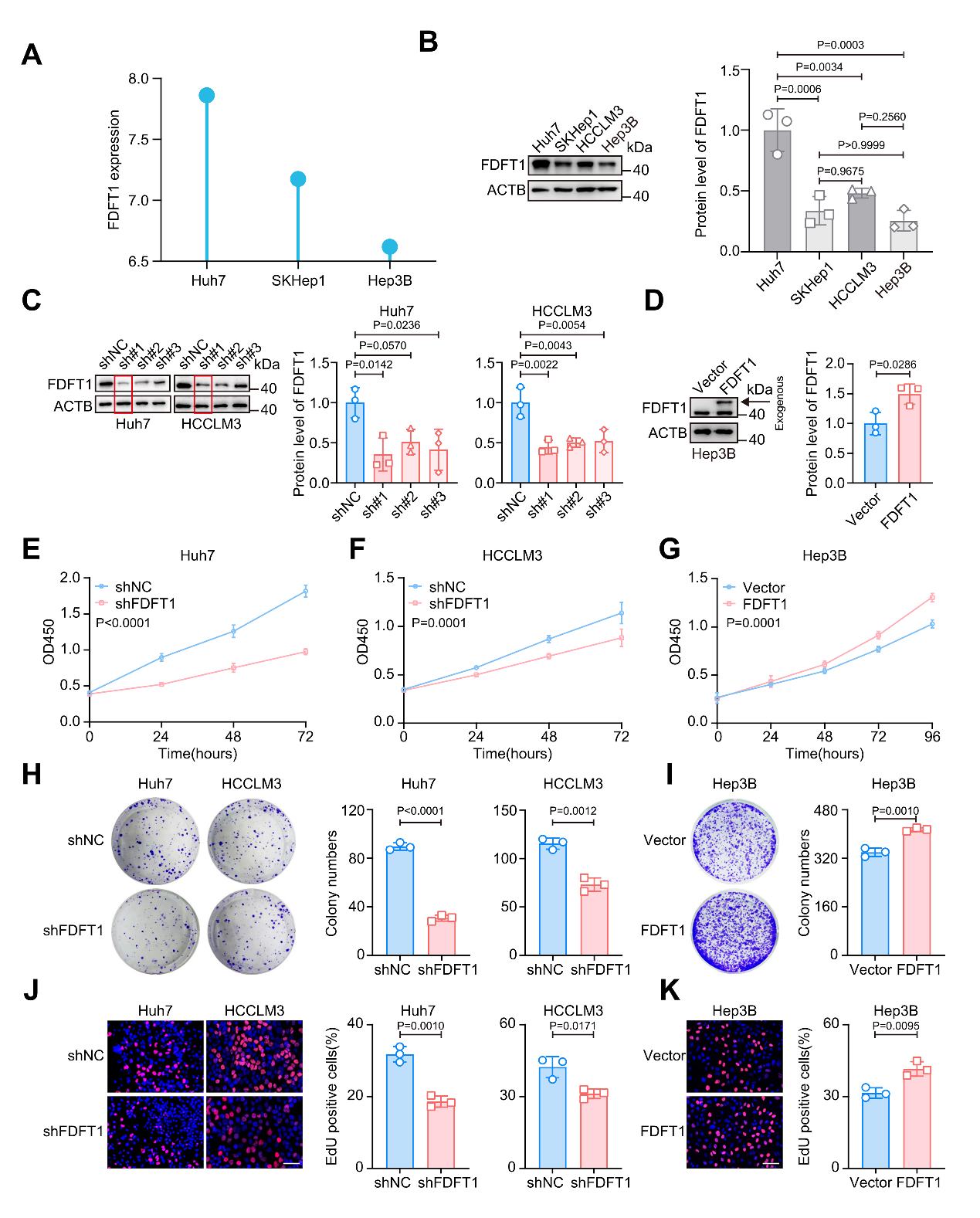


Figure S2. FDFT1 promotes HCC proliferation *in vitro*. A) The mRNA expression levels of FDFT1 in HCC cells (Huh7, SKHep1 and Hep3B) from the CCLE database. B) The protein expression levels of FDFT1 in HCC cells (Huh7, SKHep1, HCCLM3 and Hep3B). n=3. C-D) Western blot results show FDFT1 knockdown (C) or overexpression (D) in HCC cells. n=3. E-G) CCK8 assays of FDFT1 knockdown (E-F) or overexpression (G) in HCC cells. H-I) Clone formation assays of FDFT1 knockdown (H) or overexpression (I) in HCC cells. n=3. J-K) EdU assays of FDFT1 knockdown (J) or overexpression (K) in HCC cells, Scale bars, 50 μm. n=3. Data are presented as mean ± SD. Images were quantified by image J (B-D). Data were analyzed by two-way (E-G) or one-way (B-C) ANOVA with Bonferroni multiple-comparison correction (E-G), and unpaired t test (D, H-K). CCLE, cancer cell line encyclopedia.

**
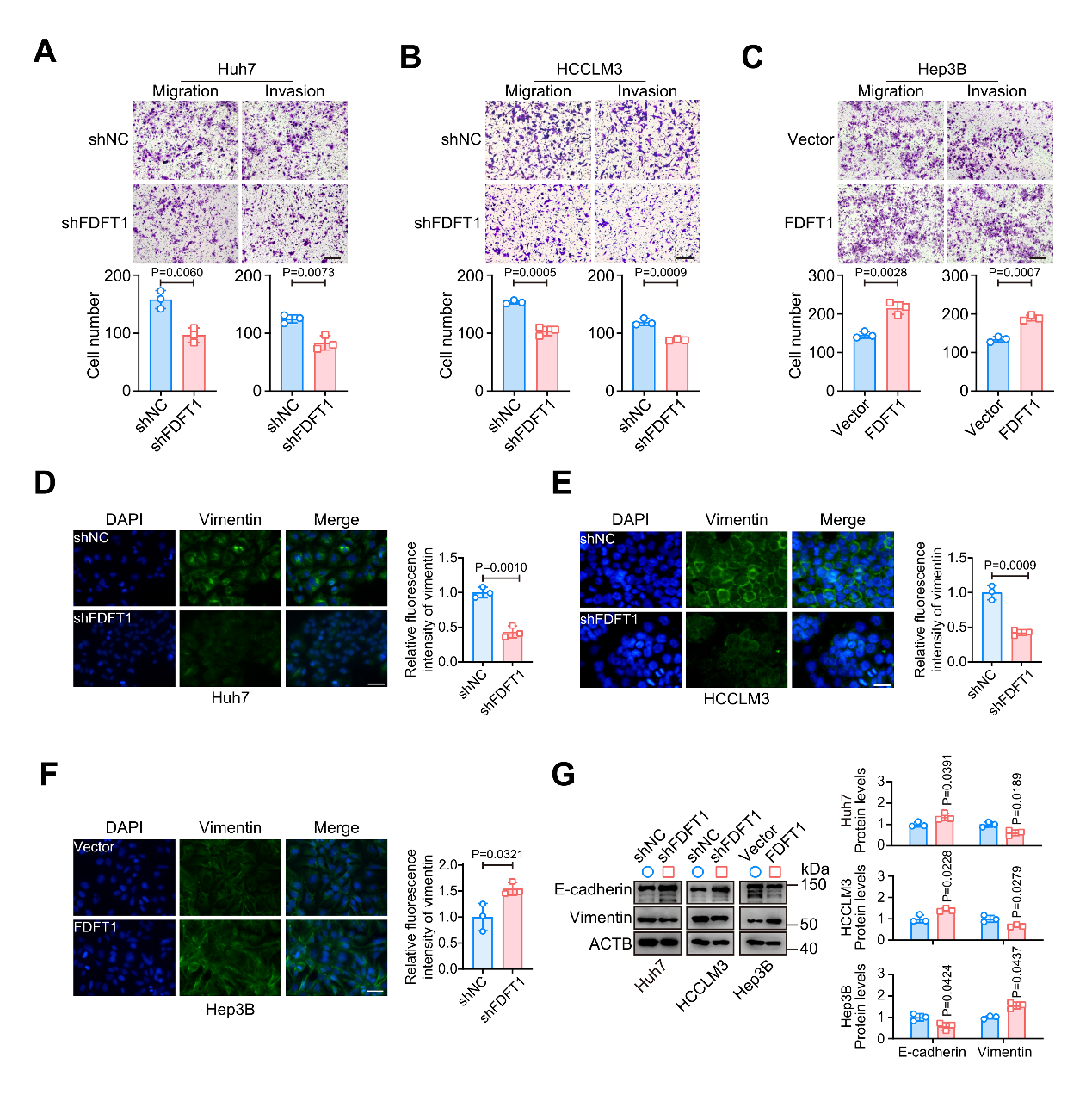
**

Figure S3. FDFT1 promotes HCC metastasis *in vitro*. A-C) Migration and invasion assays of FDFT1 knockdown (A-B) or overexpression (C) in HCC cells. Count was completed through image J (A-C). Scale bars, 100 μm. n=3. D-F) Representative images of vimentin immunofluorescence in FDFT1 knockdown (D-E) and overexpression (F) cells. Scale bars, 25 μm. n=3. G) Western blot of E-cadherin and vimentin in FDFT1 knockdown and overexpression cells. n=3. Data are presented as mean ± SD. Images were quantified by image J (D-G). Data were analyzed by unpaired t test (A-G).

**
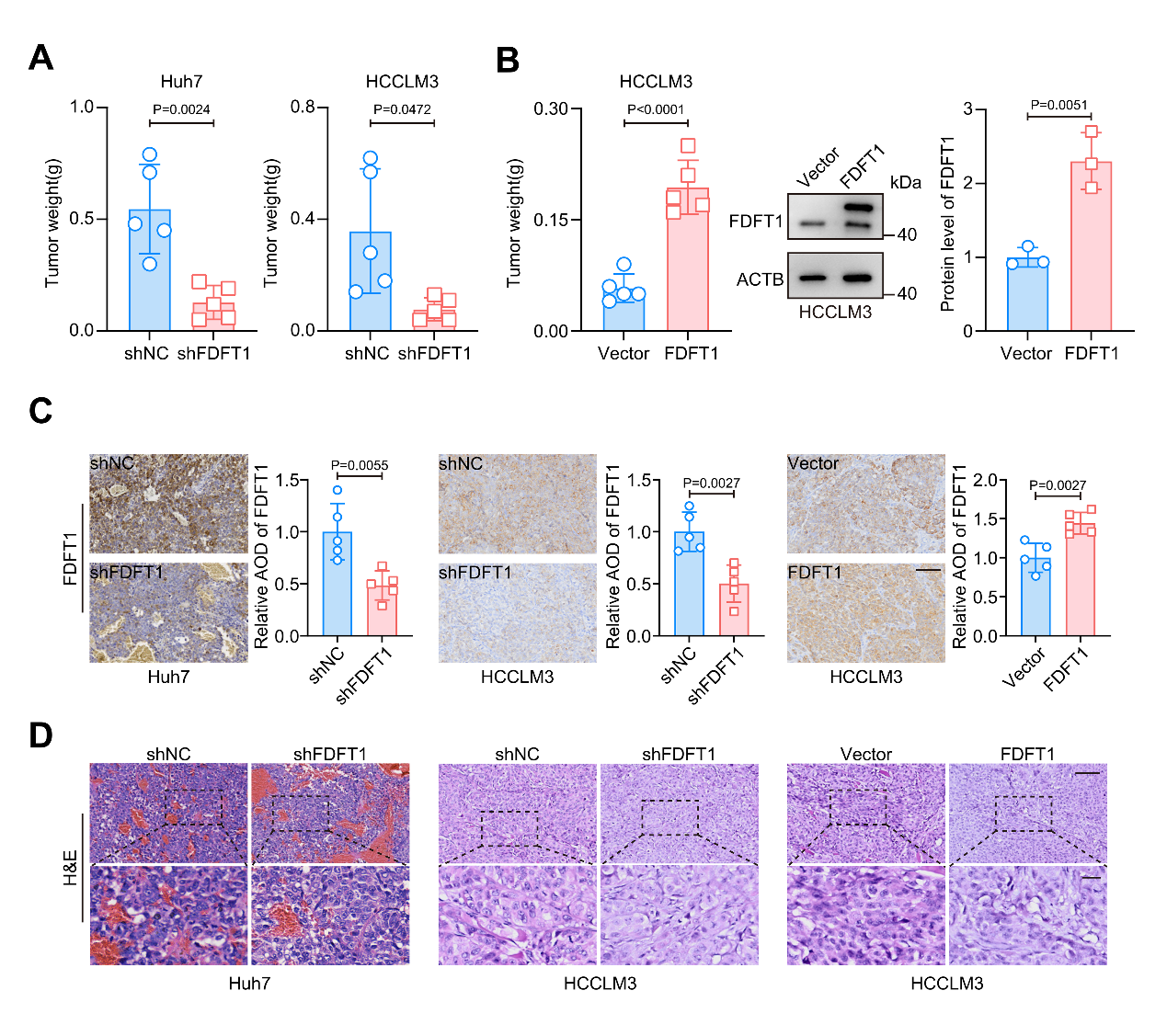
**Figure S4. FDFT1 knockdown inhibits HCC proliferation *in vivo*. A-B) Tumor weight of FDFT1 knockdown (A) or overexpression (B-left panel) in subcutaneous xenograft models. Western blot results show FDFT1 overexpression in HCCLM3 cells (B-right panel, n=3). n=5. C-D) Representative images of FDFT1 (C) or H&E (D) IHC in subcutaneous xenograft models. Scale bars, 100 (C, D-upper panel) and 25 (D-lower panel) μm. n=5. All Data are presented as mean ± SD. Images were quantified by image J (B-right panel, C). Data were analyzed by unpaired t test (A-C). H&E, hematoxylin-eosin; AOD, average optical density. **
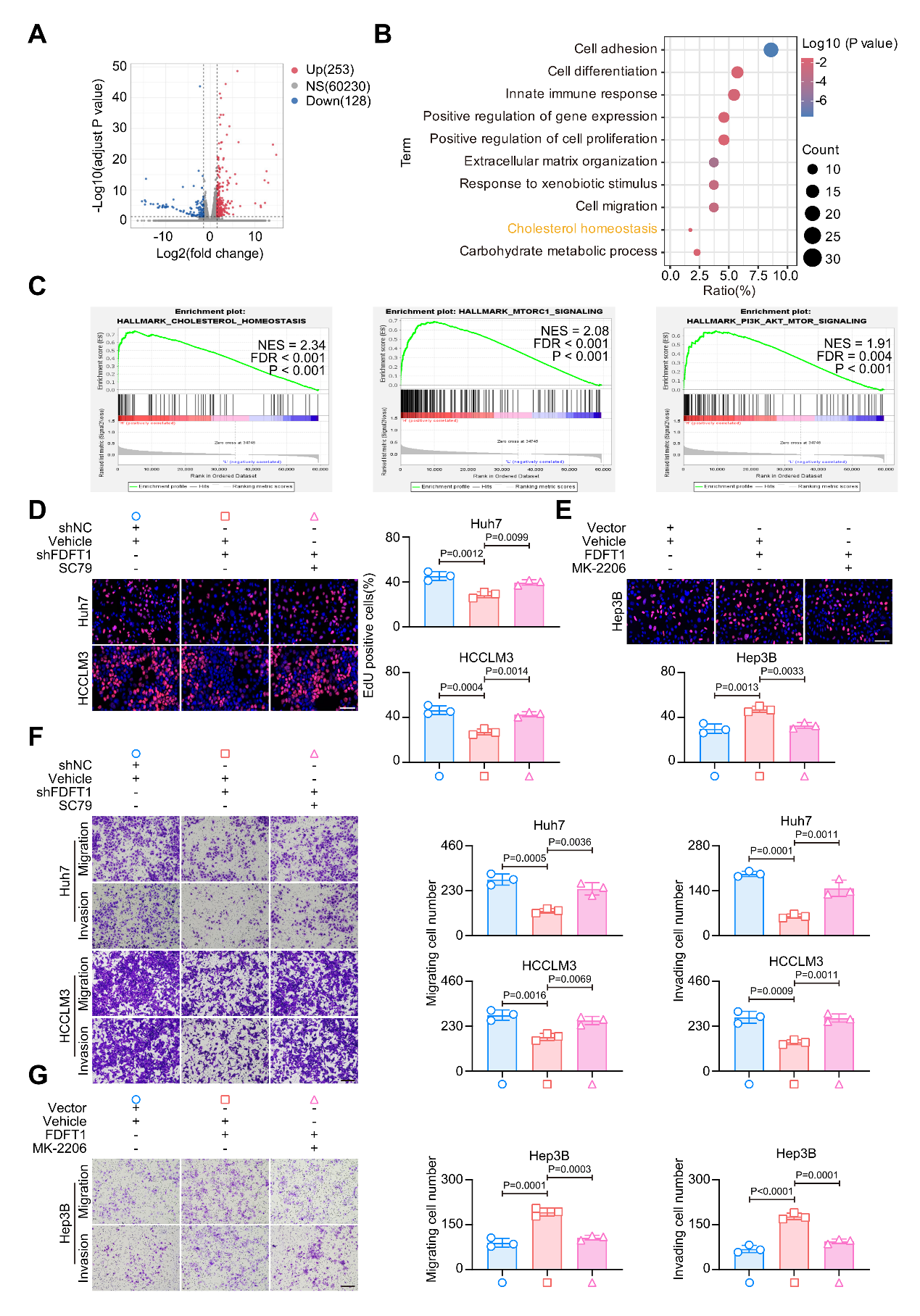
**

Figure S5. FDFT1 knockdown affects cholesterol homeostasis, AKT signaling and regulates the HCC phenotypes via AKT1. A-B) Volcano plot (A) and GO-BP analysis (B) of DEGs between shFDFT1 and shNC groups in Huh7 cells. C) GSEA analysis between high and low FDFT1 expression in the TCGA HCC cohort. D-E) EdU assays show that FDFT1 regulates HCC proliferation partially depending on AKT1. Scale bars, 50 μm. n=3. F) Migration and invasion assays show that FDFT1 knockdown inhibits HCC metastasis partially depending on AKT1. Scale bars, 100 μm. n=3. G) Migration and invasion assays show that FDFT1 overexpression promotes HCC metastasis partially depending on AKT1. Scale bars, 100 μm. n=3. All Data are presented as mean ± SD. Images were quantified by image J (D-G). Cells were treated with MK-2206 (50 nM) or SC79 (5 μM) for 24h (D-G). Data were analyzed by one-way ANOVA (D-G) with Bonferroni multiple-comparison correction. GO-BP, gene ontology-biological process; GSEA, gene set enrichment analysis; DEGs, differentially expressed genes.


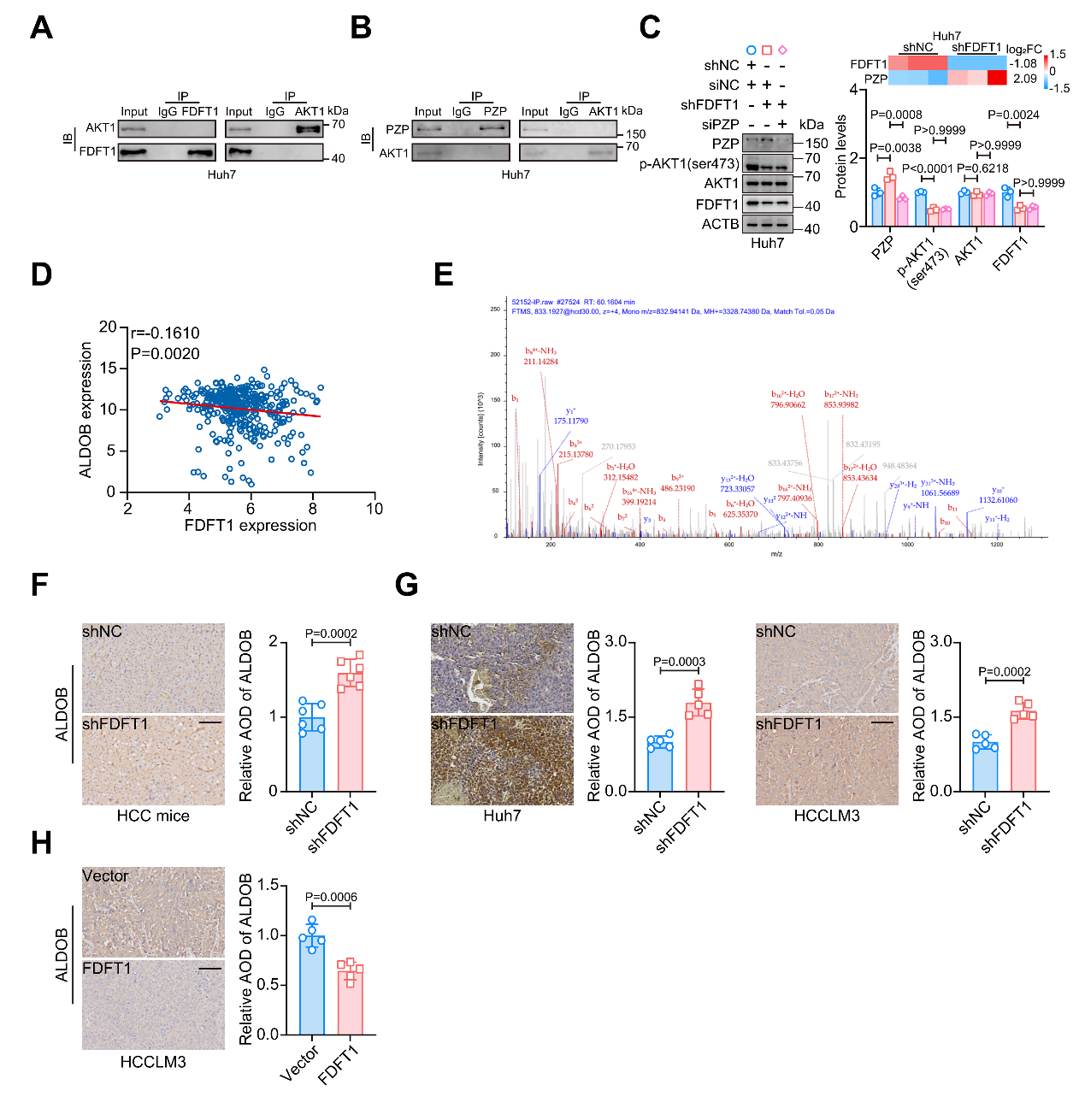


Figure S6. FDFT1 knockdown promotes ALDOB expression. A) Co-IP results show that FDFT1 does not interact with AKT1 in Huh7 cells. B) Co-IP results show that PZP does not interact with AKT1 in Huh7 cells. C) Western blot results show that PZP knockdown does not rescue the downregulation of AKT1 phosphorylation caused by FDFT1 knockdown (left, right-down panels) and sequencing results show that PZP mRNA expression is up-regulated after FDFT1 knockdown (right-upper panel). n=3. D) Correlation between ALDOB and FDFT1 in the TCGA cohort. E) Mass spectrogram of the peptides from ALDOB interacting with AKT1 in Huh7 cells. F) Representative images of ALDOB IHC in FDFT1 knockdown primary HCC models. Scale bars, 100 μm. n=6. G-H) Representative images of ALDOB IHC in FDFT1 knockdown (G) or overexpression (H) subcutaneous xenograft models. Scale bars, 100 μm. n=5. All Data are presented as mean ± SD. Images were quantified by image J (C, F-H). Data were analyzed by one-way ANOVA (C) with Bonferroni multiple-comparison correction and unpaired t test (F-H). Correlation analysis was completed by Spearman correlation analysis (D). Co-IP, coimmunoprecipitation; AOD, average optical density.

**
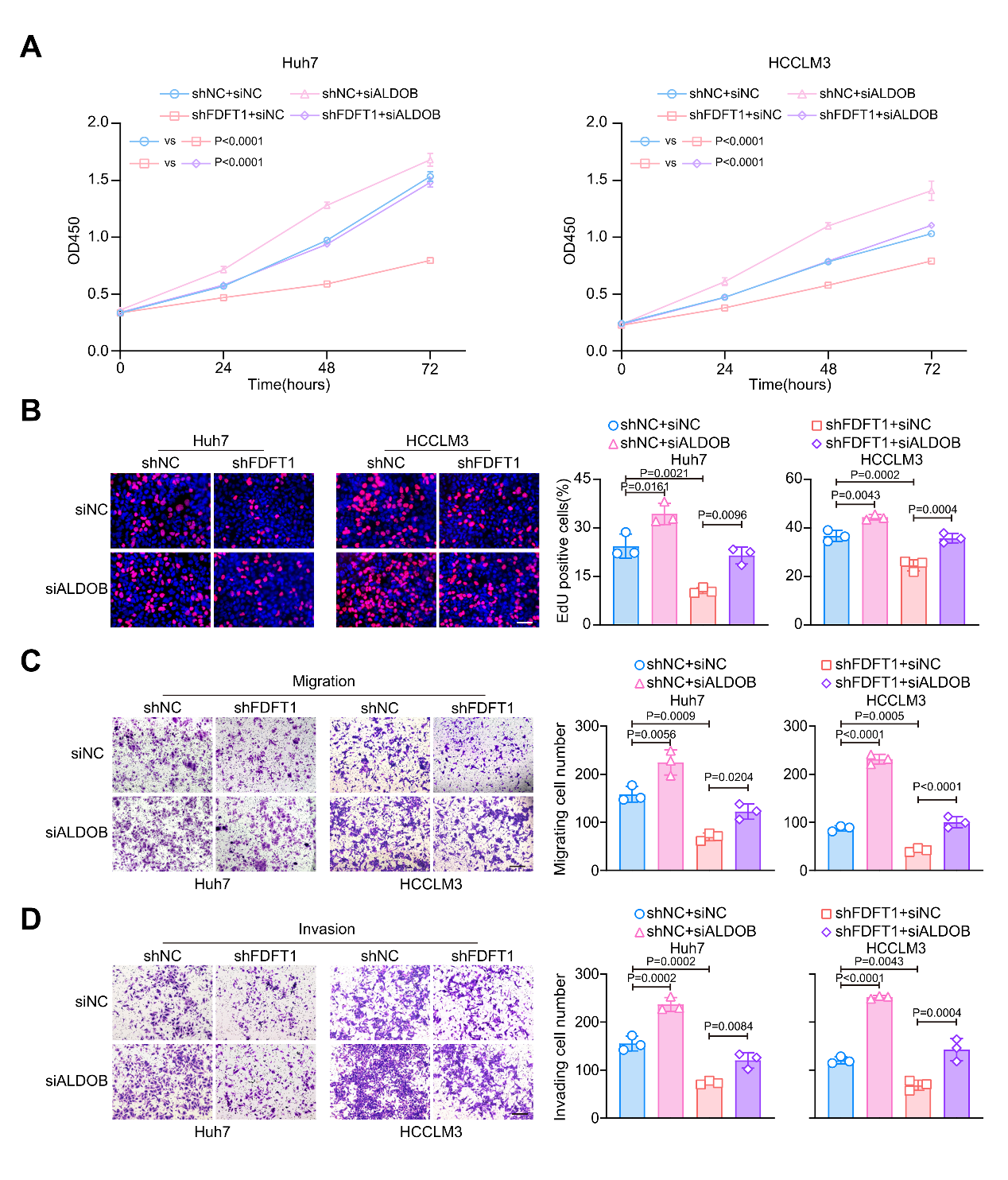
**

Figure S7. FDFT1 knockdown inhibits proliferation and metastasis via ALDOB in vitro. A-B) CCK8 (A) and EdU (B) assays show that ALDOB knockdown using siRNA promotes HCC proliferation and restores inhibition of proliferation caused by FDFT1 knockdown in HCC cells. Count was completed through image J. Scale bars, 100 μm. n=3. C-D) Migration (C) and invasion (D) assays show that ALDOB knockdown using siRNA promotes HCC metastasis and rescues inhibition of metastasis induced by FDFT1 knockdown. Count was finished by Image J. Scale bars, 100 μm. n=3. All Data are presented as mean ± SD and were analyzed by two-way (A) or one-way ANOVA (B-D) with Bonferroni multiple-comparison correction. siRNA, small interfering RNA.


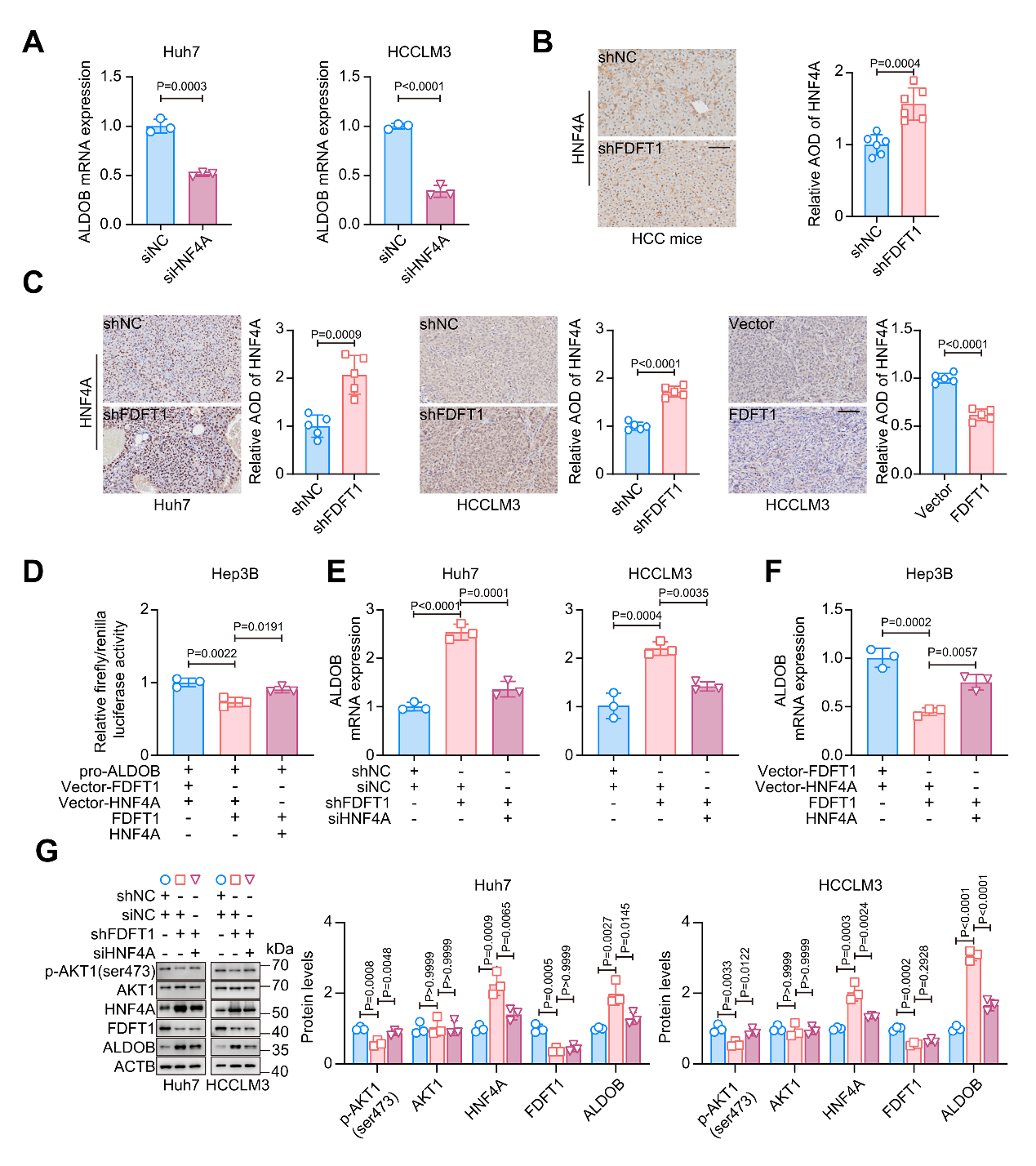


Figure S8. FDFT1 knockdown inhibits ALDOB expression via HNF4A. A) The mRNA levels of ALDOB in FDFT1 knockdown cells. n=3. B) Representative images of HNF4A IHC in liver specific FDFT1 knockdown mice. Scale bars, 100 μm. n=6. C) Representative images of HNF4A IHC in subcutaneous xenograft models. Scale bars, 100 μm. n=5. D) Dual-luciferase reporter assays of HNF4A overexpression in FDFT1 overexpression cells. n=3. E-F) The mRNA levels of ALDOB suggest that FDFT1 regulates ALDOB expression via HNF4A. n=3. G) Western blot assays show that HNF4A knockdown rescues HNF4A upregulation, ALDOB upregulation and p-AKT1 (ser473) downregulation induced by FDFT1 knockdown. n=3. All data are presented as mean ± SD. Images were quantified by image J (B-C, G). Data were analyzed by unpaired t test (A-C) and one-way (D-G) ANOVA with Bonferroni multiple-comparison correction. pro-ALDOB, promoter of ALDOB; AOD, average optical density.


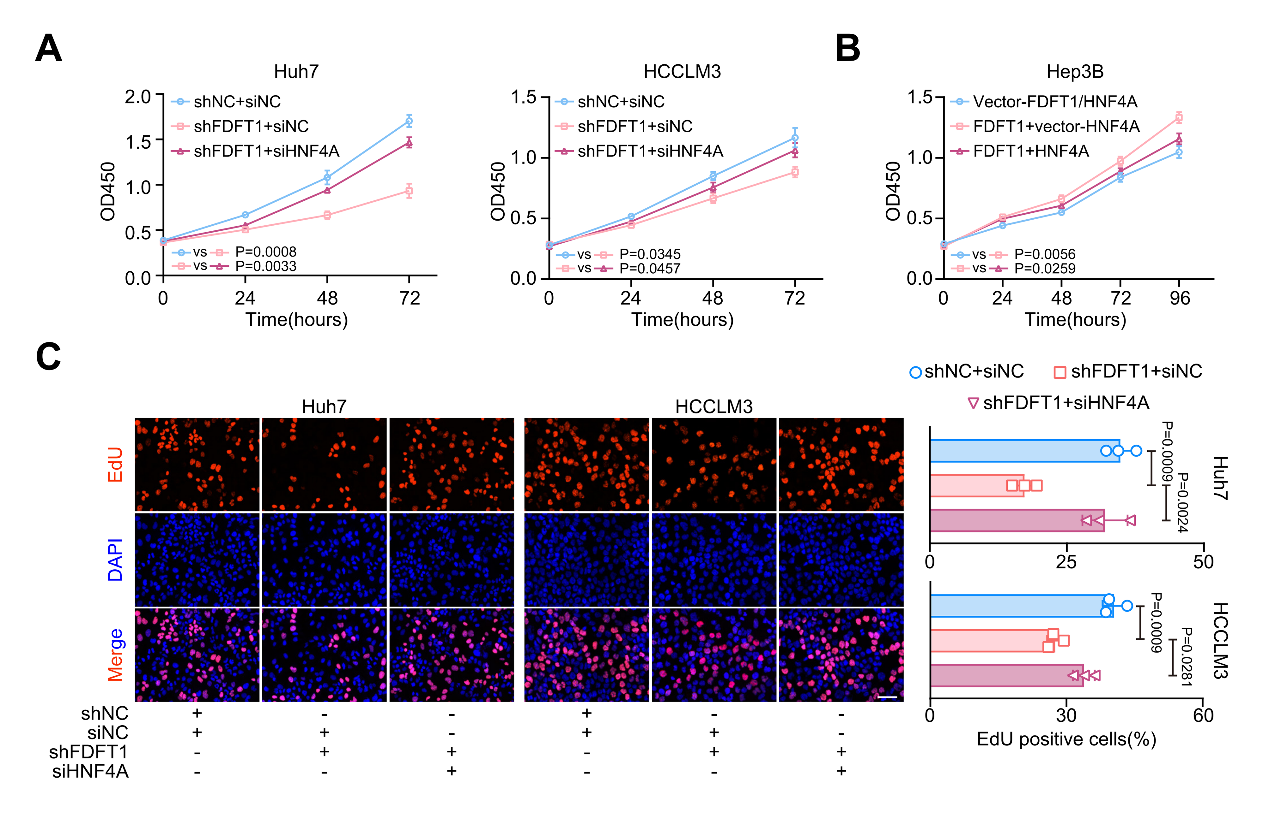


Figure S9. FDFT1 knockdown inhibits proliferation via HNF4A in vitro. A-B) CCK8 assays show that FDFT1 knockdown (A) or overexpression (B) regulate HCC proliferation partially depending on HNF4A. n=3. C) EdU assays show that FDFT1 regulates HCC proliferation partially depending on HNF4A. Scale bars, 50 μm. n=3. All data are presented as mean ± SD. Images were quantified by image J (C). Data were analyzed by one-way (C) or two-way (A-B) ANOVA with Bonferroni multiple-comparison correction.


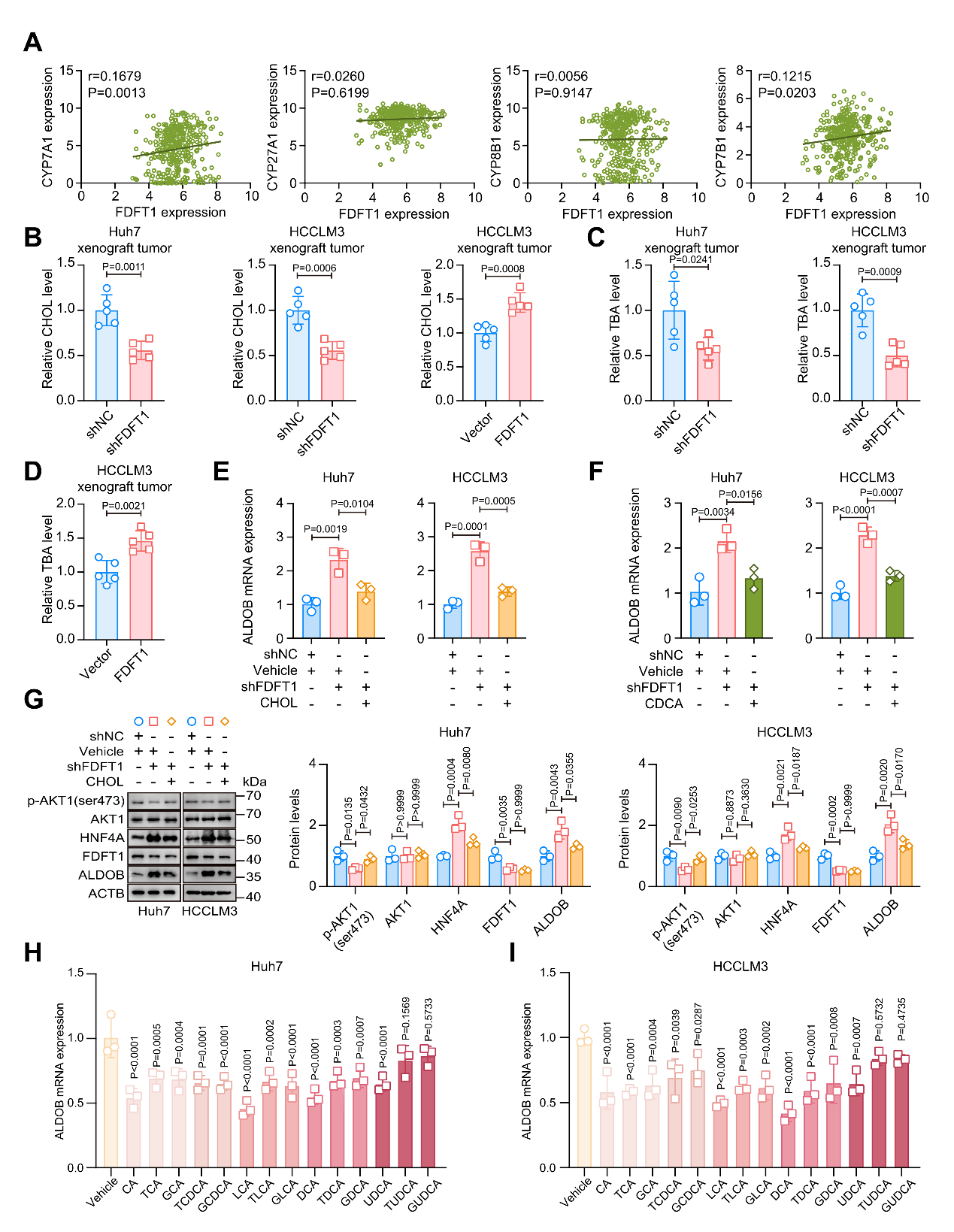


Figure S10. FDFT1 knockdown inhibits HCC via the cholesterol/bile acid axis. A) Correlation between FDFT1 and other enzymes involved in bile acid synthesis in the TCGA cohort. B) The relative CHOL levels of tumor tissues from xenograft models constructed by FDFT1 knockdown or overexpression. n=5. C-D) The relative TBA levels of tumor tissues from xenograft models constructed by FDFT1 knockdown (C) or overexpression (D). n=5. E-F) The mRNA levels of ALDOB show that adding cholesterol (E) or CDCA (F) restores ALDOB expression in FDFT1 knockdown cells. n=3. G) Western blot shows that adding cholesterol restores AKT1 phosphorylation and ALDOB expression in FDFT1 knockdown cells. n=3. H-I) The mRNA levels of ALDOB in Huh7 (H) or HCCLM3 (I) cells treated with various bile acids. All groups were compared with control group vehicle. n=3. Cells were treated with cholesterol (10 μg/ml), YM-53601 (5 μM) or various indicated bile acids (100 μM) for 24 h. All data are presented as mean ± SD. Images were quantified by image J (G). Data were analyzed by Spearman correlation analysis (A), unpaired t test (B-D) and one-way (E-I) ANOVA with Bonferroni multiple-comparison correction. CHOL, cholesterol; CDCA, chenodeoxycholic acid; TBA, total bile acid; CA, cholic acid; TCA, taurocholic acid; GCA, glycocholic acid; TCDCA, taurochenodeoxycholic acid; GCDCA, glycochenodeoxycholic acid; LCA, lithocholic acid; TLCA, taurolithocholic acid; GLCA, glycolithocholic acid; DCA, deoxycholic acid; TDCA, taurodeoxycholic acid; GDCA, glycodeoxycholic acid; UDCA, ursodeoxycholic acid; TUDCA, tauroursodeoxycholic acid; GUDCA, glycoursodeoxycholic acid.

**
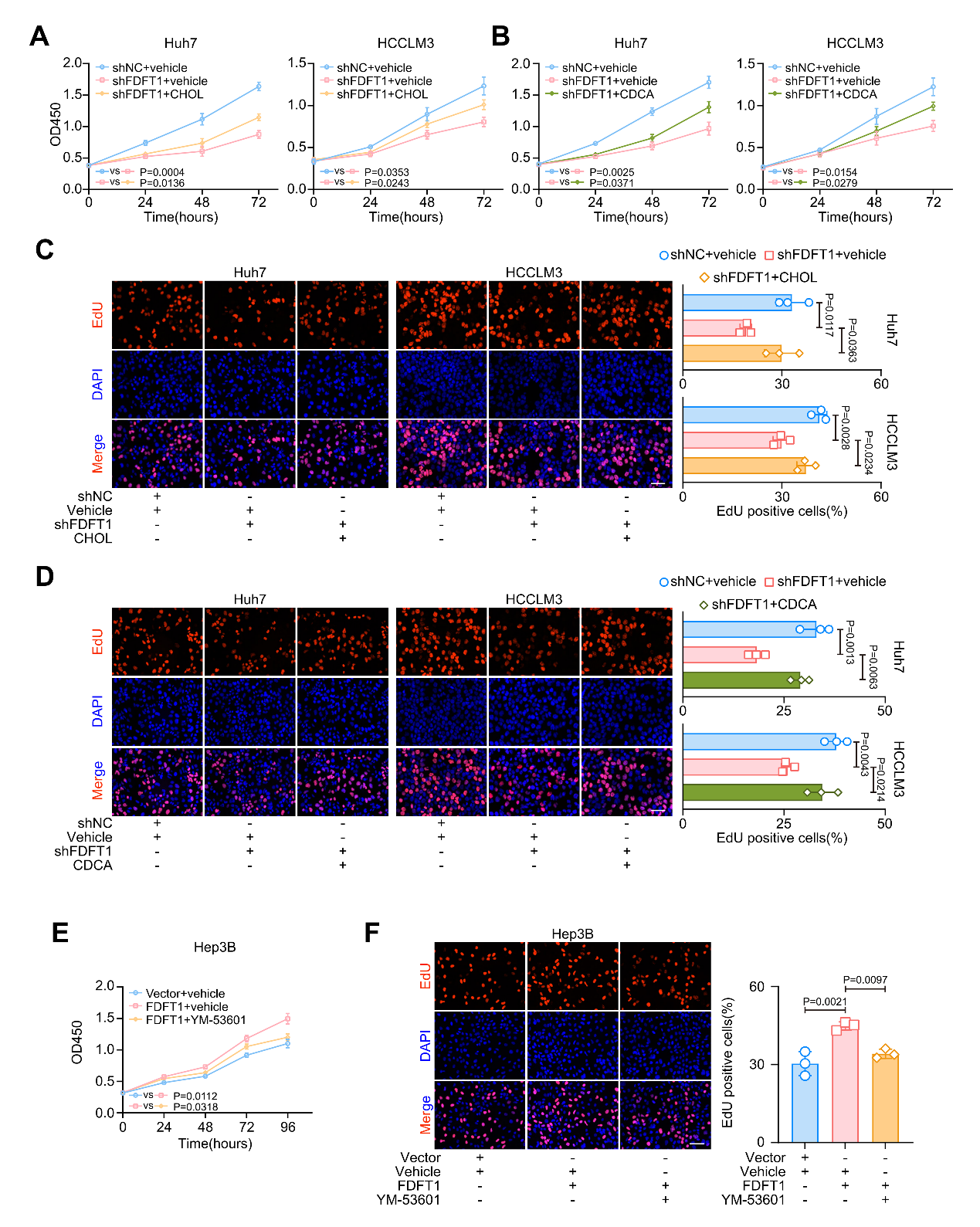
**

Figure S11. FDFT1 knockdown inhibits HCC proliferation via the cholesterol/bile acid axis. A-B) CCK8 assays show that adding cholesterol (A) or CDCA (B) partially restores cell viability in FDFT1 knockdown cells. n=3. C-D) EdU assays show that adding cholesterol (C) or CDCA (D) restores cell proliferation in FDFT1 knockdown cells. Scale bars, 50 μm. n=3. E-F) CCK8 (E) and EdU (F) assays show that adding YM-53601 partially rescues cell proliferation in FDFT1 overexpression cells. Scale bars, 50 μm. n=3. Cells were treated with cholesterol (10 μg/ml), CDCA (100 μM) or YM-53601 (5 μM) for 24 h. All data are presented as mean ± SD. Images were quantified by image J (C-D, F). Data were analyzed by one-way (C-D, F) or two-way (A-B, E) ANOVA with Bonferroni multiple-comparison correction. CHOL, cholesterol; CDCA, chenodeoxycholic acid.

**
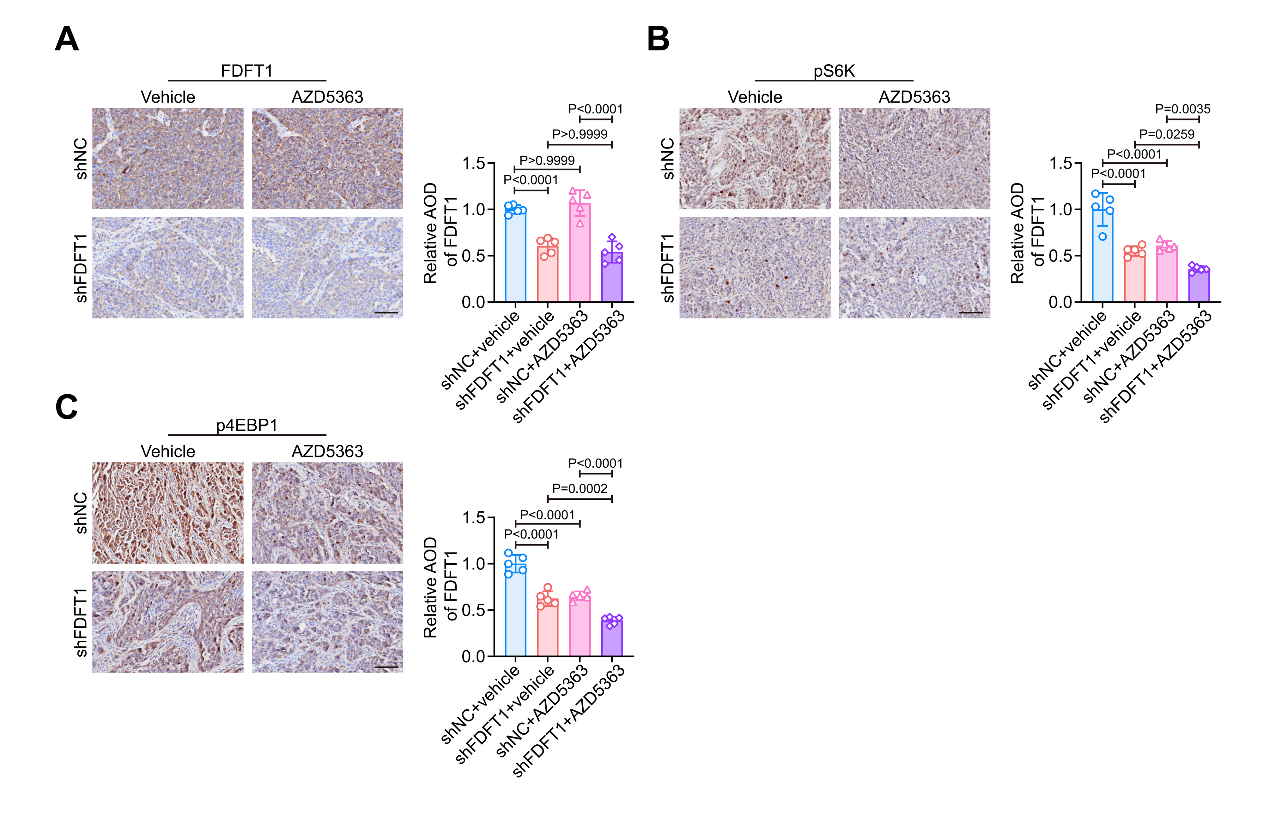
**

Figure S12. Representative images of IHC in subcutaneous xenograft tumors. A) Representative IHC images of FDFT1. Scale bars, 100 μm. n=5. B) Representative IHC images of pS6k. Scale bars, 100 μm. n=5. C) Representative IHC images of p4EBP1. Scale bars, 100 μm. n=5. All data are presented as mean ± SD. Images were quantified by image J. Data were analyzed by one-way ANOVA with Bonferroni multiple-comparison correction (A-C).

**Table S1. Clinical features between low and high FDFT1 HCC patients (n=90)**

| Variables |  | Low FDFT1(n=36) | High FDFT1(n=54) | P value |
| --- | --- | --- | --- | --- |
| Age |  |  |  |  |
|  | > 60 | 16 | 22 | 0.727 |
|  | ≤ 60 | 20 | 32 |  |
| Gender |  |  |  |  |
|  | male | 22 | 37 | 0.469 |
|  | female | 14 | 17 |  |
| Tumor size |  |  |  |  |
|  | > 5 cm | 9 | 27 | 0.018 |
|  | ≤ 5 cm | 27 | 27 |  |
| BCLC stage |  |  |  |  |
|  | B-C | 7 | 23 | 0.022 |
|  | 0-A | 29 | 31 |  |
| Tumor number |  |  |  |  |
|  | multiple | 5 | 13 | 0.710 |
|  | solitary | 31 | 41 |  |
| Distant metastasis |  |  |  |  |
|  | present | 3 | 15 | 0.024 |
|  | absent | 33 | 39 |  |
| Cirrhosis |  |  |  |  |
|  | with | 4 | 11 | 0.248 |
|  | without | 32 | 43 |  |
| HbsAg |  |  |  |  |
|  | positive | 8 | 15 | 0.554 |
|  | negative | 28 | 39 |  |

Data were analyzed by Chi-square test.

**Table S2. shRNA and siRNA sequences**

| Target | Sequence |
| --- | --- |
| shNC | TTCTCCGAACGTGTCACGT |
| shFDFT1-#1 | ACCTGTCGTTTGTCATGCTTT |
| shFDFT1-#2 | CCTACCTTTCGAGACTCAGAA |
| shFDFT1-#3 | ACAAACATCATCCGTGACTAT |
| siALDOB | ACCCUCUACCAGAAGGACA |
| siHNF4A | TCAACGACCGCCAGTATGA |
| siPZP | CTACCAGTATCTCGGTTAA |
| shFDFT1-mouse | GCAGCTTGAAGACCTCTACATAGTGAAGCC-ACAGATGTATGTAGCAGGTCTTCAAGCTGCT |
| shNC-mouse | AAGGCAGAAGTATGCAAAGCATTAGTGAAGC-CACAGATGTAATGCTTTGCATACTTCTGCCTG |

**Table S3. Antibodies information used in the study**

| Antibody | Catalogue # | Company | Dilution |
| --- | --- | --- | --- |
| ACTB (β-actin) | EM21002 | HUABIO | 1:2000 (WB) |
| FDFT1 | HA720048 | HUABIO | 1:2000 (WB), 1:100 (IHC, IF),  1 μg/sample (IP), |
| Vimentin | ET1610-39 | HUABIO | 1:2000 (WB), 1:100 (IF) |
| E-cadherin | ET1607-75 | HUABIO | 1:2000 (WB) |
| Ki67 | 27309-1-AP | Proteintech | 1:10000 (IHC) |
| ALDOB | 18065-1-AP | Proteintech | 1:4000 (WB), 1:200 (IHC, IF),  1 μg/sample (IP) |
| AKT1 | ET1609-47 | HUABIO | 1:2000 (WB),  1 μg/sample (IP) |
| pAKT1 (ser473) | ET1607-73 | HUABIO | 1:2000 (WB), 1:100 (IHC) |
| HNF4A | ET1611-43 | HUABIO | 1:2000 (WB), 1:100 (IHC, IF),  10 μg/sample (CHIP) |
| Rabbit IgG | HA1002 | HUABIO | 1 μg/sample (IP),  10 μg/sample (CHIP) |
| Goat anti-rabbit IgG | ZB-2301 | ZSGB-BIO | 1:10000 (WB) |
| Goat anti-mouse IgG | ZB-2305 | ZSGB-BIO | 1:10000 (WB) |
| p4EBP1 | R22929 | Zenbio | 1:100 (IHC) |
| pS6 | HA721803 | HUABIO | 1:1000 (IHC) |
| PZP | 21742-1-AP | Proteintech | 1:3000 (WB),  1 μg/sample (IP) |

**Table S4. Real-time quantitative reverse transcription PCR (qRT-PCR) primer information used in the study**

| Gene | Primer |
| --- | --- |
| ALDOB | Forward: GAAGTGGCGTGCTGTGCTGAG |
|  | Reverse: CTGCTGACAGATGCTGGCGTAG |
| EGR1 | Forward: AGTTACCCCAGCCAAACCAC |
|  | Reverse: GGTCATGCTCACTAGGCCAC |
| ECT2 | Forward: TTGATTTTCCTCTTCCAGTCCTTA |
|  | Reverse: CCAAGCTAGTCCTCCCAGTAG |
| CCND1 | Forward: GAAGGAGACCATCCCCCTGA |
|  | Reverse: CAATGAAATCGTGCGGGGTC |
| SLC2A2 | Forward: TGGAGCTCTCTTGATGGGGT |
|  | Reverse: ACGATGGCCAGCTGATGAAA |
| OTC | Forward: ACCAAGCTGTTGCTGACAAA |
|  | Reverse: CACTTCTTCTGGCTTTCTGGG |
| APOPA5 | Forward: CTTTCCGCCAGGACACCTAC |
|  | Reverse: TTGTTGAAACTCTGGGGCGA |
| GAPDH | Forward: GGAGTCCACTGGCGTCTTCA |
|  | Reverse: GTCATGAGTCCTTCCACGATACC |
| Primer for Chip-qPCR | Forward: AGGGGTGTTGATGGCTTAGTTCA |
|  | Reverse: CCAGTCCTCCAAAACCTAGCTG |
